# Cost-effectiveness analysis of secukinumab versus other biologics and apremilast in the treatment of active Psoriatic arthritis: a Finnish perspective

**DOI:** 10.1186/s12962-018-0162-3

**Published:** 2018-11-16

**Authors:** Timo Purmonen, Kari Puolakka, Devarshi Bhattacharyya, Minal Jain, Janne Martikainen

**Affiliations:** 1 Novartis Finland Oy, Espoo, Finland; 2 South Karelia Social and Health Care District, Lappeenranta, Finland; 3 Novartis Product Life Cycle Services-NBS, Novartis Healthcare Private Limited, Hyderabad, India; 4 School of Pharmacy, University of Eastern Finland, Kuopio, Finland

**Keywords:** Psoriatic arthritis, Cost-effectiveness, Biologics, Secukinumab, Incremental cost effectiveness ratio, Quality adjusted life years

## Abstract

**Objective:**

To study cost-effectiveness of an interleukin (IL)-17A inhibitor secukinumab, with other biologics and apremilast in patients with Psoriatic arthritis (PsA) from payer perspective in Finland.

**Methods:**

In this semi-Markov model, subcutaneous (SC) secukinumab was compared with SC treatments etanercept and its biosimilar, certolizumab pegol, adalimumab and its biosimilar, golimumab, ustekinumab, intravenous (IV) treatment infliximab, as well as oral non-biologic apremilast. Patients without prior exposure (naïve) to biologics and without moderate to severe psoriasis were considered for secukinumab 150 mg group. Secukinumab 300 mg group included naïve patients with moderate to severe psoriasis and all patients with prior biologic exposure. The PsA Response Criteria (PsARC) at 12-week was primary criteria for treatment response. Other clinical as well as cost related model inputs were derived from relevant clinical trials as well as Finnish publications. The key model outcomes were quality-adjusted life years and incremental cost-effectiveness ratio. An annual 3% discount rate was applied to all future costs and benefits. Model input variations were assessed through sensitivity analyses and alternative scenario analyses.

**Results:**

For a lifetime horizon (60 years), secukinumab 150 mg dominated all branded SC biologics and apremilast with highest QALY of 8.01 and lowest lifetime cost of €187,776, while it was cost-effective against IV infliximab among biologic-naïve patients without moderate to severe psoriasis. Secukinumab 300 mg was cost-effective against all branded SC biologics and apremilast and dominated IV infliximab among biologic-naïve patients with moderate to severe psoriasis, while it was cost-effective in biologic experienced patients. With the one-way sensitivity analysis, PsARC response, drug acquisition cost, and health assessment questionnaire score were the most important parameters affecting the outcomes. Across all treatment groups, patients on secukinumab were most likely to achieve highest net monetary benefit than other competitors in probabilistic sensitivity analysis. With alternative scenario analysis, results largely remained unchanged.

**Conclusions:**

Secukinumab is a cost-effective treatment for PsA patients from a Finnish payer’s perspective.

**Electronic supplementary material:**

The online version of this article (10.1186/s12962-018-0162-3) contains supplementary material, which is available to authorized users.

## Introduction

Psoriatic arthritis (PsA), a disease associated with psoriasis, is a chronic, multiform inflammation of joints, entheses and tendons but can also affect spine [1–4]. There are a lot of variations in the disease severity. Potentially, inflammation in PsA can lead to irreversible structural changes in joints and bones [5]. Incidence rates ranging from 10 to 23.1 per 100,000 person-years have been reported in local epidemiological studies [6, 7]. Painful joints and entheses limit patients’ daily activities, which along with psoriatic skin involvement, causes substantial lowering of health-related quality of life [8]. In addition, PsA is associated with comorbidities, especially cardiovascular diseases, and poses significant economic burden over healthcare systems [9].

The European League against Rheumatism (EULAR) recommends the usage of conventional synthetic disease-modifying anti-rheumatic drugs (csDMARDs) as a first-line therapy for PsA patients. Patients with failure of or inadequate response to csDMARD should be considered for biologics like tumor necrosis factor Inhibitors (TNFi) and subsequently the interleukin inhibitors (IL 12/23 or IL 17 inhibitors) [10, 11]. The EULAR recommends the use of synthetic DMARD like apremilast in patients whom biologics are not appropriate. The Group for Research and Assessment of Psoriasis and Psoriatic arthritis (GRAPPA) recommends to start treatment with csDMARDs followed by TNFi and IL inhibitors, if patients have inadequate response to previous therapy. However, in case of enthesitis, dactylitis, or nail psoriasis, the GRAPPA recommends TNFi and IL inhibitors without a trial with csDMARDs [12, 13]. In line with the EULAR and the GRAPPA guidelines, the Finnish current care guidelines also recommend starting the therapy with csDMARD (methotrexate) and switch to TNFi if such treatment is not tolerated or the response to treatment is inadequate. If TNFi are ineffective, switching to IL-17 inhibitors like secukinumab is recommended [14].

Secukinumab is a first-in-class, recombinant, high-affinity, fully human, monoclonal antibody that neutralizes IL-17A [4, 15]. In 2015, the European Medicines Agency approved secukinumab 300 mg for the treatment of active PsA as subcutaneous injection for patients with concomitant moderate to severe psoriasis or inadequate response to TNFi and secukinumab 150 mg for all other patients [16]. In multiple phase III placebo-controlled trials (FUTURE trials and their extensions), secukinumab showed rapid, significant, and sustained efficacy across various clinical domains with a favorable safety profile in biologic-naïve as well as in biologic-experienced patients (failure with up to three TNFi) [17–23]. In a recent network meta-analysis, secukinumab was found to be the most efficacious and safe treatment for PsA among all licensed IL-inhibitor biologics [24]. In addition, a matching-adjusted indirect comparison (MAIC) [25] established better efficacy of secukinumab against adalimumab. An ongoing head-to-head trial comparing secukinumab against adalimumab (EXCEED) [26] is aimed to confirm results obtained from the MAIC.

This study reports the results of a cost-effectiveness analysis of subcutaneous (SC) secukinumab versus currently licensed biologics, biosimilars (SC certolizumab pegol, etanercept and its biosimilar, adalimumab and its biosimilar, golimumab, ustekinumab and intravenous [IV] infliximab) and oral non biologic apremilast in Finland.

## Methods

### Patient population and interventions

The cost-effectiveness analysis was conducted in adult PsA patients of at least 18 years of age, with active disease in spite of treatment with NSAIDs, csDMARDs, and/or TNFi. The baseline patient characteristics were derived from the FUTURE 2 study [17], as shown in Additional file 1: Table S1. Patients were classified into three groups based on prior exposure and response to biologics and psoriasis severity as, (1) biologic-naïve (no prior exposure to biologics) without moderate to severe psoriasis, (2) biologic-naïve with moderate to severe psoriasis, and (3) biologic-experienced (having prior exposure to biologics). Biologic-naïve patients without moderate to severe psoriasis received 150 mg of secukinumab, while biologic-naïve patients with moderate to severe psoriasis and biologic-experienced patients received secukinumab 300 mg. The secukinumab dosing regimen was based on the approved marketing authorization guidelines [16] and is reflective of the common prescription practice of rheumatologists in Finland. All patients are assumed to continue receiving concomitant standard of care (SoC) treatments. Information related to treatment regimens like dosing and frequencies are provided in Additional file 1: Table S2.

### Model structure

**Fig. 1:**
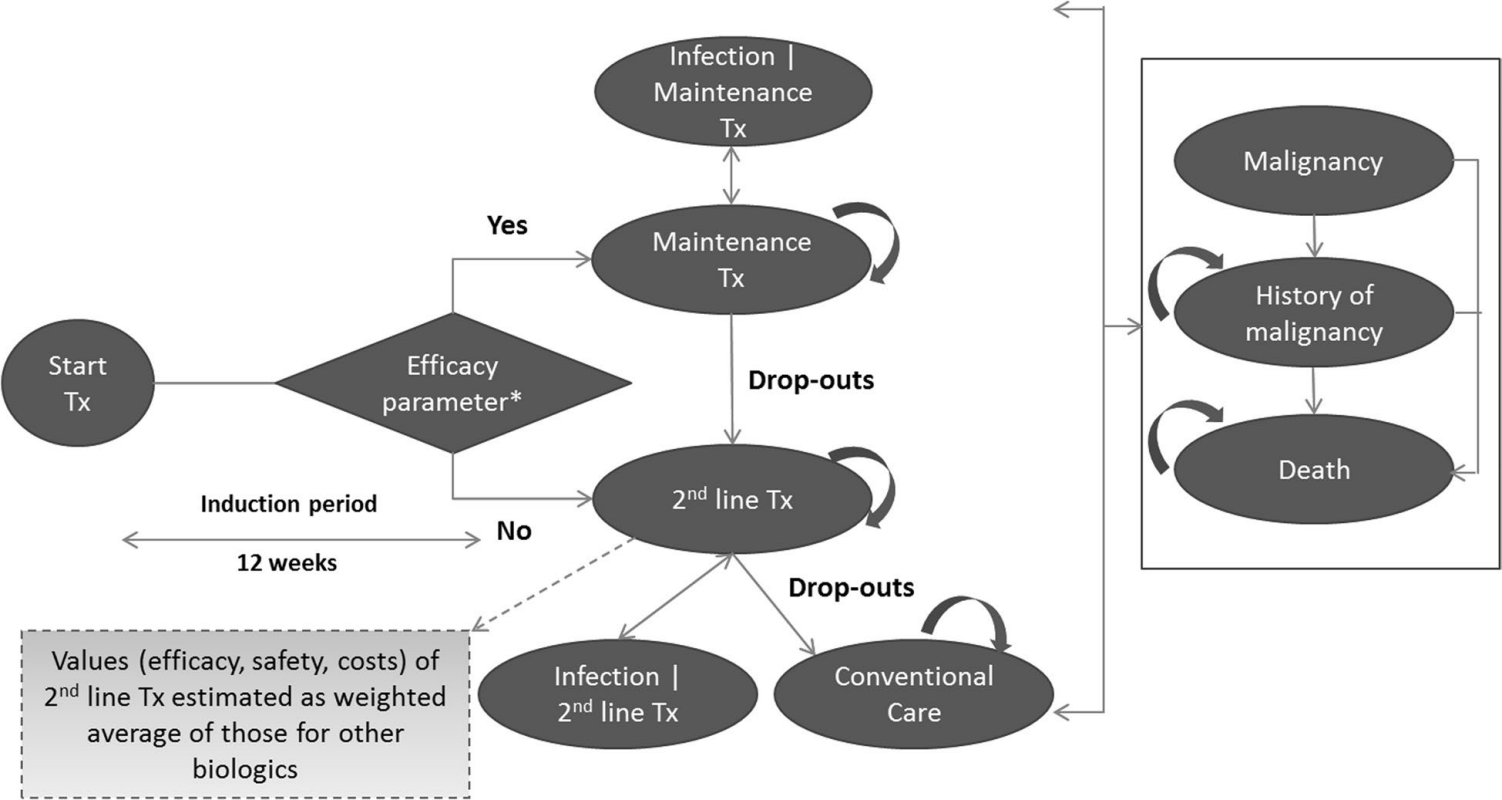
Model structure. *The efficacy parameter depends on the criteria chosen—PsARC alone (for base case analysis), PASI, or a combination of PsARC and PASI (for alternative scenario analysis). Tx, treatment

The model, as shown in Fig. 1, had a semi-Markov structure. This model is adapted from the recently published cost-effectiveness study of secukinumab in Canada [27]. Similar structure was used in the York model [28, 29] as well as other economic analyses of TNFi treatments for PsA [30–34]. This model also allowed assessment of the impact of adverse events, such as malignancy and tuberculosis, on mortality.

Treatment initiation defined the entry point for patients, while a 3-month duration was considered as induction period. At the end of the induction period, patients were evaluated for treatment response using the PsA response criteria (PsARC). The PsARC has been used extensively as a response criteria in similar analyses, including the York model, which allows comparability of this analysis with prior publications [28, 29, 31–34]. The PsARC as a treatment response criteria is also accepted by the National Institute of Health and Care Excellence (NICE) of the UK [35]. Based on treatment response, probabilities of withdrawal, serious infection including tuberculosis, malignancy, and death, patients transitioned to different health states (Fig. 1). Patients within the “Malignancy” state were assumed to be at a higher mortality risk for 5 years into that state.

Patients were able to withdraw from their initial biologic and transition to a subsequent/second line biologic as per preference of patients and/or physicians. As data on the effectiveness of subsequent biologics and treatment switches were limited, average values of efficacy, costs, treatment withdrawal rates, and adverse event rates were used for all biologics. Patients receiving subsequent biologics were assumed to continue on this treatment or move to the SoC if they dropped out.

### Model inputs

#### Clinical inputs

The 3-month results for PsARC response, the Psoriasis Area and Severity Index (PASI) response, and change from baseline in the Health Assessment Questionnaire (HAQ) were the main clinical inputs used in the model, as shown in Table 1. Due to the lack of head-to-head trials in the biologic-naïve population, comparative effectiveness data were obtained from a network meta-analysis (NMA) assessing PsARC and PASI outcomes [36], as shown in Additional file 1: Figures S1, S2.

**Table 1:** Clinical inputs at 3 months

	SEC 150	SEC 300	CER P	ETN	ADA	INF	GOL	UST	APR
PsARC response [36] (% of patients)									
*Biologic Naïve* ^*a*^	59.82%	51.18%	73.61%	70.91%	62.84%	78.65%	79.96%	73.61%	73.61%
*Biologic experienced* ^*b*^	N/A	82.45%	67.89%	68.38%	51.72%	67.73%	59.90%	60.87%	46.04%
PASI response [36] (% of patients)									
PASI score	*Biologic Naïve* ^*c*^								
PASI < 50	24.70%	21.94%	26.30%	26.30%	30.00%	13.35%	39.43%	26.30%	26.30%
PASI 50-74	22.73%	21.92%	23.12%	23.12%	23.79%	17.84%	24.31%	23.12%	23.12%
PASI 75-89	23.00%	23.39%	22.71%	22.71%	21.90%	23.22%	19.23%	22.71%	22.71%
PASI 90-99	29.57%	32.76%	27.87%	27.87%	24.31%	45.58%	17.03%	27.87%	27.87%
PASI 100	0.00%	0.00%	0.00%	0.00%	0.00%	0.00%	0.00%	0.00%	0.00%
	*Biologic experienced* ^*d*^								
PASI < 50	N/A	19.63%	74.10%	74.48%	58.98%	48.84%	63.39%	58.00%	80.40%
PASI 50-74	N/A	16.87%	11.80%	11.72%	12.47%	8.83%	12.86%	12.31%	10.11%
PASI 75-89	N/A	23.01%	8.91%	8.76%	13.96%	14.30%	12.64%	14.17%	6.47%
PASI 90-99	N/A	40.49%	5.20%	5.04%	14.60%	28.03%	10.82%	15.52%	3.01%
PASI 100	N/A	0.00%	0.00%	0.00%	0.00%	0.00%	0.00%	0.00%	0.00%
Change in HAQ^*e*#^									
*Biologic Naïve*									
Regardless of PsARC response	− 0.4780	− 0.5780	− 0.2410	− 0.5259	− 0.3579	− 0.5578	− 0.3629	− 0.1988	− 0.2363
Given PsARC response	− 0.5430	− 0.6880	− 0.3950	− 0.6400	− 0.4900	− 0.6600	− 0.4400	− 0.3950	− 0.3950
Given no PsARC response	− 0.2310	− 0.2500	0.0000	− 0.2000	− 0.1400	− 0.2000	− 0.0600	0.0000	0.0000
*Biologic experienced* ^*e*^									
Regardless of PsARC response	N/A	− 0.5360	− 0.2410	− 0.5259	− 0.3579	− 0.5578	− 0.3629	− 0.1988	− 0.2363
Given PsARC response	N/A	− 0.5360	− 0.3950	− 0.6400	− 0.4900	− 0.6600	− 0.4400	− 0.3950	− 0.3950
Given no PsARC response	N/A	− 0.3270	0.0000	− 0.2000	− 0.1400	− 0.2000	− 0.0600	0.0000	0.0000

ADA, adalimumab; APR, apremilast, CER P, certolizumab pegol; ETN, etanercept; GOL, golimumab; INF, infliximab; PsARC, Psoriatic arthritis response criteria; SEC 150, secukinumab 150 mg; SEC 300, secukinumab 300 mg; UST, ustekinumab

^a^Biologic-naive data for certolizumab pegol and ustekinumab were not available and were assumed equivalent to be average of other biologics in the NMA. Trials for etanercept 50 mg once weekly did not link into the network, thus data for etanercept 25 mg twice weekly were used. Data for experienced population was lacking and was computed by applying a reduction to mixed population. Secukinumab: 0.43% reduction, other Tumor Necrosis Factor-alpha inhibitor (TNFi): 10.1%

^b^Biologic-experienced data for ustekinumab were not available and were assumed equivalent to average of other biologics in the NMA. Trials for etanercept 50 mg once weekly did not link into the network, thus data for etanercept 25 mg twice weekly were used

^c^Biologic-naive data for certolizumab pegol, etanercept, golimumab and ustekinumab were not available and were assumed equivalent to the average of other biologics in the network meta-analysis (NMA). Secukinumab 150 mg was evaluated in biologic-naïve population without moderate to severe psoriasis and secukinumab 300 mg was evaluated in biologic-naïve patients with moderate to severe psoriasis

^d^Data for experienced population was lacking and was computed by applying a reduction to mixed population. For Sec and other Tumor Necrosis Factor-alpha inhibitor (TNFi): PASI 50: 1.34% and 43.08% reduction, PASI 75: 6.84% and 40.64% reduction, PASI 90: 6.67% and 41.86%

^e^Secukinumab 150 mg was evaluated in biologic-naïve population without moderate to severe psoriasis and secukinumab 300 mg was evaluated in biologic-naïve patients with moderate to severe psoriasis. For biologic-experienced patients, data were assumed equivalent to that of mixed population

^#^Sources: Biologic experienced, regardless of PsARC response—SEC 150 and SEC 300 from FUTURE 2 trial; CER P set equal to placebo; ETN, ADA, INF, GOL, UST, and APR estimated from changes in HAQ with and without PsARC response and PsARC response probabilities; biologic experienced, given PsARC response—SEC 150, SEC 300 from FUTURE 2 trial; CER P, UST, and APR set equal to placebo; ETN, ADA, INF, and GOL from Cawson et al. [31]; biologic experienced, given no PsARC response—SEC 150 and SEC 300 from FUTURE 2 trial; CER P, UST, and APR set equal to placebo; ETN, ADA, INF, and GOL from Cawson et al. [31]; Biologic naïve, regardless of PsARC response—SEC 150, SEC 300, and placebo from FUTURE 2 trial; CER P, ETN, ADA, INF, GOL, UST, and APR set equal to biologic naïve or experienced, regardless of PsARC response; biologic naïve, given PsARC response—SEC 150, SEC 300 from FUTURE 2 trial; CER P, ETN, ADA, INF, GOL, UST, and APR set equal to biologic naïve or experienced, given PsARC response; biologic naïve, given no PsARC response—SEC 150 and SEC 300 from FUTURE 2 trial; CER P, ETN, ADA, INF, GOL, UST, APR set equal to biologic naïve or experienced, given no PsARC response

Clinical efficacy in biologic-experienced patients is expected to be lower than that for biologic-naïve patients [37]. Hence clinical efficacy data in biologic-experienced patients were calculated by applying adjustment rates to available clinical data that compared biologic-naïve to biologic-experienced patients [17, 38]. Treatment withdrawals rates are provided in Additional file 1: Table S3. Wherever clinical data for a comparator were missing, average of relevant data from other comparators within similar population were used as a substitute.

#### Cost and resource use

Four types of costs were included: drug acquisition costs, disease-related costs, medical support costs, and adverse event costs, as shown in Table 2 and Additional file 1: Tables S4, S5. Wherever required, these costs were inflation adjusted with the latest available index and converted to Euro (€).

**Table 2:** Costs inputs

Drug/input	Cost (€)	Unit
*Drug acquisition costs* ^*a*^		
SEC 150 mg	584.43	Per dose
SEC 300 mg	1168.86	Per dose
CER P 200 mg	483.07	Per prefilled syringe
ETN 50 mg	260.04	Per prefilled syringe
ADA 40 mg	527.41	Per prefilled syringe
INF 100 mg	436.06	Per vial
GOL 50 mg	1086.93	Per prefilled syringe
UST 45/90 mg	3124.96	Per prefilled syringe
APR 10–30 mg	418.52	Per 14 day pack
APR 30 mg	14.93	Per tablet
ETN biosimilar 50 mg	182.02	Per prefilled syringe
ADA biosimilar 40 mg	369.19	Per prefilled syringe
MTX 7.5 mg	0.51	Per dose
*Disease-related costs*		
Intercept	297.89	Per 3 months
Cost per HAQ change	131.61	Per 1-unit change per 3-months
*Health states*		
Uncontrolled psoriasis (PASI < 75)	253.14	Per 3 months
Controlled psoriasis (PASI ≥ 75)	20.46	Per 3 months

ADA, adalimumab; APR, apremilast; CER P, certolizumab pegol; ETN, etanercept; GOL, golimumab; HAQ, Health Assessment Questionnaire; INF, infliximab; LEF, leflunomide; MTX, methotrexate; PASI, Psoriasis Area Severity Index; SEC, secukinumab; SUL, sulfasalazine; UST, ustekinumab

Source: Costs from the York model (Rodgers et al., [28]) who extracted costs from the following sources. Cost for a 1-point change in HAQ obtained from Kobelt et al. [49]. Cost for uncontrolled psoriasis obtained from Department of Health [50]. Cost for controlled psoriasis obtained from Hartman et al. [51]

^a^Finnish medicinal products and prices database [accessed on-line 2.1.2018]. All prices exclude value added tax. Retail price for SC products, and wholesale price for IV products is applied according to local guidelines. Infliximab price is weighed with market share. A cost of €382 (2016 value) is added for each IV administration [48] Biosimilar pricing for etanercept and adalimumab assumed to be 30% less than brand; they were not available in the market at the time of analyses, and thus only used for sensitivity analysis

The acquisition costs for all brand drugs were obtained from the Finnish medicinal products and prices database. Biosimilar prices for etanercept and adalimumab were considered 30% less than that for the brand price. Due to unavailability of Finnish PsA related costs, this category of costs were retrieved from the published York model in PsA (Table 2) [28]. A linear regression method based on the change from baseline in HAQ scores was employed to obtain the arthritis-related costs. The psoriasis-related costs were based on PASI response, taking into account both uncontrolled (PASI < 75) and controlled (PASI ≥ 75) disease states. Costs related to medical support and adverse events were based on national healthcare unit costs and were confirmed by expert advice (Additional file 1: Tables S4, S5).

The indirect costs were not included in the base case analysis. However, alternative scenarios were run by incorporating indirect costs in a form of productivity loss due to PsA. Loss of productivity was calculated based on Health Assessment Questionnaire Disability Index (HAQ-DI) scores. Based on the HAQ-DI scores, patients were distributed into six categories, and unemployment rate was assigned to each category: rate 24% for HAQ-DI score < 0.6, rate 41% for score ≥ 0.6–1.1, rate 45% for score ≥ 1.1–1.6, rate 44% for score ≥ 1.6–2.1, rate 68% for score ≥ 2.1–2.6, rate 87% for rate ≥ 2.6–3.0 [39]. The unemployment rates were multiplied with the cost of productivity derived from the Finnish National Institute of Health and Welfare to obtain productivity loss due to unemployment for each category (Additional file 1: Table S5).

#### Other inputs

In the base case analysis, utility values from the FUTURE 2 trial [17] were used for each cycle, which were calculated by converting EQ-5D scores with Dolan [40] algorithm using a representative UK population. Utility values for scenario analysis were taken from the York model, [28] which were calculated by using HAQ and PASI scores and applying linear regression method. The values are shown in Additional file 1: Table S6. Three mortality risks were included in the analysis as shown in Additional file 1: Table S7. Finnish age-specific life tables were used to take into account the all-cause mortality. The disease-specific mortality risk was based on risk values used in the York model [28]. The adverse event-related mortality risk is based on the evidence showing similar mortality risk for PsA patients to that of the general population [41].

### Base case analysis

The base case analysis compared cost-effectiveness of secukinumab 150 mg and 300 mg with that of licensed biologics, their biosimilars, and apremilast from a Finnish payer’s perspective. Using quality-adjusted life years (QALYs) as the primary effectiveness outcome, secukinumab’s dominance over other comparators was assessed. Dominance was defined as having higher QALY at lower cost over a comparator. In case secukinumab was non-dominant, the incremental cost-effectiveness ratio (ICER) was reported. A commonly referred willingness-to-pay (WTP) threshold of €30,000/QALY was used for assessing the cost-effectiveness, as there is no mandated WTP threshold in Finland. Annual discount rates of 3% were applied for both future costs and outcomes. The analysis was done for a lifetime horizon of 60 years.

### Sensitivity analyses

Three sensitivity analyses assessed the impact of variations of input parameters on the base case results. In one-way deterministic analysis, input parameters likely to have the greatest impact on the results (e.g., PsARC response rate, HAQ change, and utility weights) were varied one at a time.

The scenario analyses allowed changing model structures and assumptions to ascertain their impact on base case results. Some of these scenarios included time horizons of 5 and 10 years, discount rates of 0% and 5%, alternative utility values, addition of indirect costs, inclusion of disutilities, and HAQ score retaining after treatment withdrawal.

In probabilistic sensitivity analysis, certain distributions were assigned for input parameters to assess the impact of sampling uncertainty. Input parameters like response rate, costs, and utility weights were varied in this analysis. The net monetary benefit (NMB) statistics was used for this analysis, as it allows simultaneous comparisons of multiple comparators [42]. NMB represents monetary value of a comparator at a defined WTP.

## Results

### Base case results

The QALYs, cost, and ICER related results for base case are provided in Table 3. For biologic-naïve patients without moderate to severe psoriasis, secukinumab 150 mg dominated all SC administered biologics, biosimilars, as well as the oral apremilast by achieving highest QALY (8.01) at lowest life-time cost (€187,776). The IV administered infliximab had slightly higher QALY (+ 0.06), but at an ICER of €680,427/QALY gained vs secukinumab. Hence infliximab was not considered as a cost-effective option compared with secukinumab 150 mg.

**Table 3:** Costs, QALYs and ICER values for all analyzed populations

Incremental	SC								IV	Oral
	SEC	ETN	ETN BS	ADA	ADA BS	CER P	GOL	UST	INF	APR
*SEC 150* *mg versus comparators in Biologic Naïve population without moderate to severe psoriasis)*										
Total cost (€)	187,776	208,375	186,581	207,568	186,543	204,899	197,566	214,749	230,650	192,319
QALYs	8.01	7.67	7.67	7.47	7.47	7.34	7.15	7.54	8.07	7.13
ICER (€/QALY)	–	Dominates	3514	Dominates	2283	Dominates	Dominates	Dominates	680,427^a^	Dominates
*SEC 300* *mg versus comparators in Naïve PsA with moderate to severe psoriasis population*										
Total cost (€)	231,477	214,225	192,431	213,555	192,530	210,480	203,855	220,313	235,354	198,422
QALYs	7.78	7.12	7.12	6.9	6.9	6.78	6.57	6.98	7.54	6.57
ICER (€/QALY)	–	25,872	49,365	20,342	37,251	20,955	22,819	13,941	Dominates	27,233
*SEC 300* *mg versus comparators in experienced population*										
Total cost (€)	256,019 €	215,772	194,424	210,940	191,975	211,230	201,556	217,194	231,440	196,293
QALYs	8.75	7.35	7.35	7.05	7.05	7.00	6.75	7.12	7.61	6.68
ICER (€/QALY)	–	28,742	40,546	26,609	34,959	25,623	27,293	23,832	21,631	28,939

ADA, adalimumab; APR, apremilast; BS, biosimilar; CER P, certolizumab pegol; ETN, etanercept; GOL, golimumab; INF, infliximab; IV, Intra Venous; PsARC, Psoriatic arthritis response criteria; QALY, quality-adjusted life-year; SEC 150, secukinumab 150 mg; SEC 300, secukinumab 300 mg; SC, subcutaneous; SoC, standard of care; UST, ustekinumab

^a^The ICER of Sec 150 mg vs. INF

In biologic-naïve patients with moderate to severe psoriasis, secukinumab 300 mg achieved the highest QALY (7.78) against all biologics, biosimilars, and oral apremilast at the life-time cost of €231,477. Based on the WTP of €30,000/QALY, secukinumab 300 mg was cost-effective against all SC branded biologics but not against the SC biosimilars of etanercept and adalimumab. Secukinumab 300 mg was cost-effective against oral apremilast, while it dominated the IV infliximab with higher QALYs and lower costs.

Biologic-experienced patients on secukinumab 300 mg achieved highest QALY (8.75) against all biologics, biosimilars, and oral apremilast at the life-time cost of €256,019. Secukinumab was cost-effective against all branded biologics and oral apremilast. When compared to etanercept and adalimumab biosimilars, the ICER was above the €30,000 WTP threshold.

**Fig. 2:**
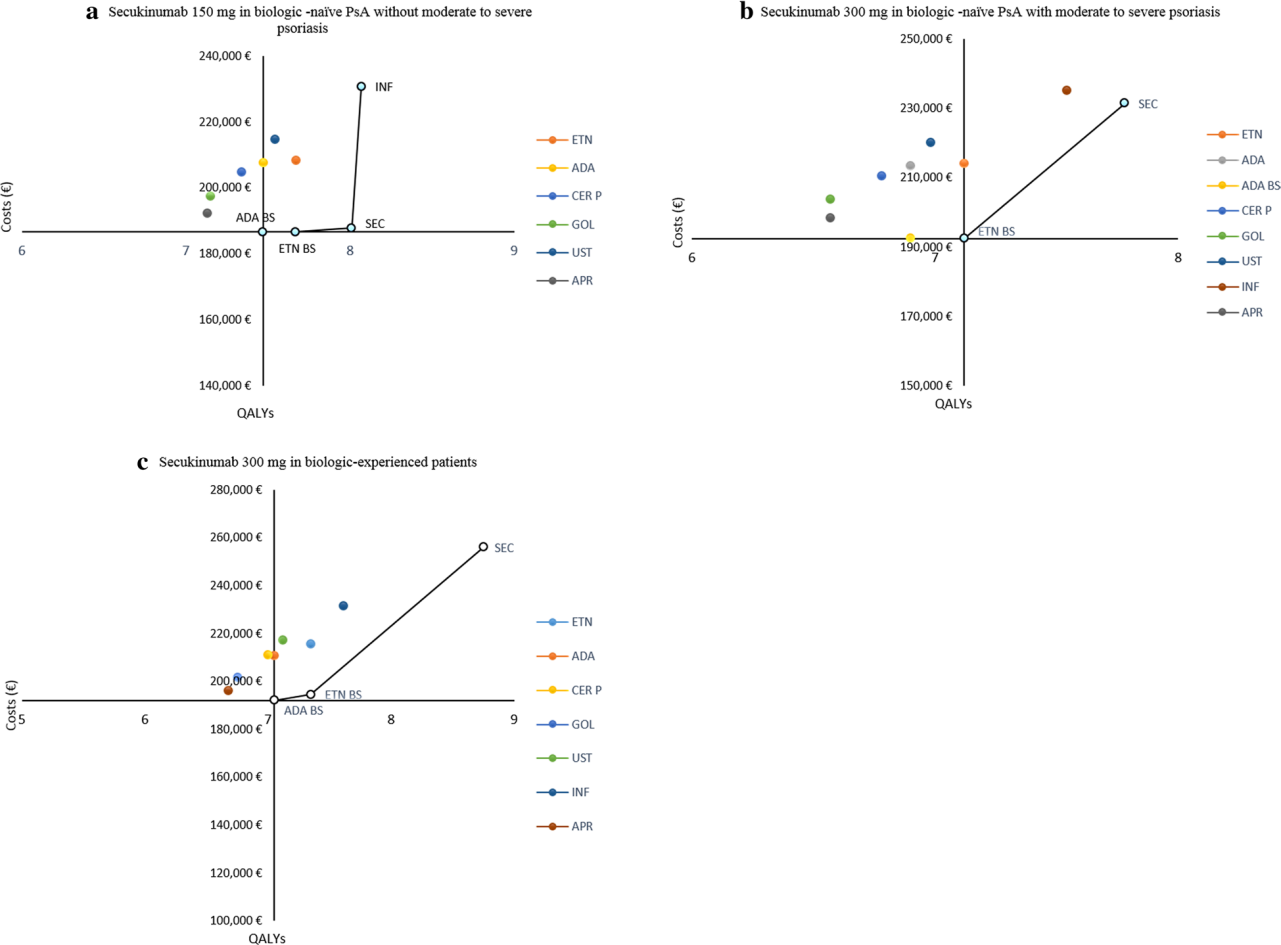
Cost effective frontier: **a** secukinumab 150 mg, **b** secukinumab 300 mg, **c** secukinumab 300 mg. ADA, adalimumab; APR, apremilast; BS, biosimilar; CER P, certolizumab pegol; ETN, etanercept; GOL, golimumab; INF, infliximab; PsA, Psoriatic arthritis; QALYs, quality adjusted life years; SEC, secukinumab; UST, ustekinumab

Multiple comparison across comparators was conducted through cost-effectiveness frontier, as seen in Fig. 2. In the biologic naïve population without moderate to severe psoriasis, secukinumab 150 mg, etanercept biosimilar, adalimumab biosimilar, and infliximab had the highest cost-effectiveness. In biologic naïve patients with moderate to severe psoriasis, etanercept biosimilar and secukinumab 300 mg were the most cost-effective options. Lastly, in biologic experienced population etanercept biosimilar, adalimumab biosimilar, and secukinumab 300 mg were the most cost-effective options. Overall, secukinumab was the only branded biologic to be cost-effective in multiple comparison across all patient groups.

#### Sensitivity analyses

In the one-way sensitivity analysis, across all patient groups, PsARC response at 3-month, drug acquisition cost, and HAQ without PsARC response were the most important input parameters that affected the expected net monetary benefits. Detailed results are shown in tornado diagrams in Additional file 1: Figures S3–S5.

Complete description of alternative inputs for different scenarios and their impact on the base case results are shown in Additional file 1: Table S8. In majority of alternative scenarios, the results were similar to that from the base case, suggesting the robustness of our analysis.

**Fig. 3:**
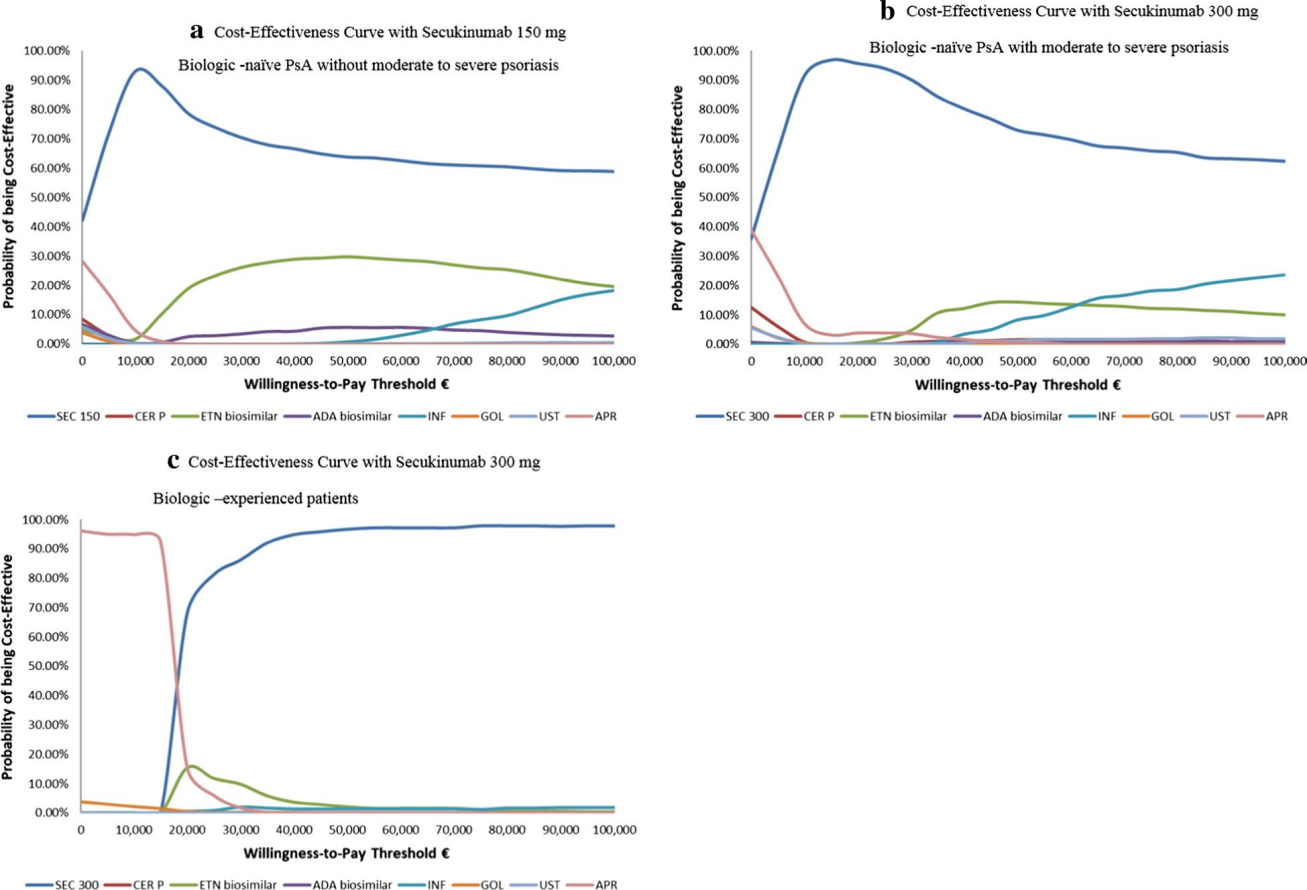
Probability of achieving highest NMB: Cost-effectiveness Acceptability Curve with **a** secukinumab 150 mg, **b** secukinumab 300 mg, **c** secukinumab 300 mg. ADA, adalimumab; APR, apremilast; CER P, certolizumab pegol; ETN, etanercept; GOL, golimumab; INF, infliximab; SEC, secukinumab; UST, ustekinumab

In the probabilistic sensitivity analysis, patients on secukinumab were most likely to achieve highest QALY compared to all other comparators. Probability of achieving highest NMB was more than 70% in biologic-naïve patients on secukinumab 150 mg at WTP of €30,000 (Additional file 1: Table S9). In patients receiving secukinumab 300 mg, irrespective of prior biologic experience, probability of achieving highest NMB was over 83% at WTP of €30,000 (Additional file 1: Table S9). The cost-effectiveness acceptability curves indicated that secukinumab had the highest probability of being cost-effective vs other comparators at the WTP threshold ranging from €20,000 to €100,000 across the three patient populations (Fig. 3). The details of the probabilistic sensitivity analysis are presented in Additional file 1: Table S9.

## Discussion

In this analysis, secukinumab proved to be either the dominant or cost-effective treatment option against all biologics and the oral apremilast among PsA patients. These results hold true in all analyzed patient populations, irrespective of their prior exposure to biologics.

With highest QALYs and lowest cost, secukinumab 150 mg provides the best economic value amongst biologic-naïve patients without moderate to severe psoriasis. Even with slightly higher QALYs (0.06), the choice of infliximab is still not justified due to its significantly higher costs (> €42,000) than secukinumab. Considering the ease of administration and lower costs, SC secukinumab may be more desirable by both patients and physicians over the IV infliximab [43, 44].

Secukinumab 300 mg was also cost-effective in both biologic-naïve (with moderate to severe psoriasis) and experienced patients compared with all branded SC biologics and oral apremilast at WTP €30,000/QALY, while it dominated the IV infliximab in these subpopulations with lower costs and higher QALYs. Secukinumab 300 mg achieved higher QALYs than biosimilars of adalimumab (biologic-naïve: + 0.88; biologic experienced: + 1.70) and etanercept (biologic-naïve: + 0.66; biologic experienced: + 1.40). However, ICER for secukinumab compared to these biosimilars were above €30,000/QALY gained.

This being a first comprehensive economic analysis of secukinumab against multiple treatment choices in Finland, will help both payers and physicians in their decision making. So far, most of such analyses have focused on the UK perspective and have largely compared etanercept to various biologics [28–34]. This analysis compares secukinumab, a newer biologic with a new mode of action, to the current standard biologic TNFi, TNFi biosimilars, as well as oral apremilast.

The strength of this analysis can be attributed to various factors. The clinical inputs in the model were taken from an NMA, consisting of 6021 patients in 20 RCTs. Patients in this NMA were a mix of those with and without prior biologic exposure, which makes clinical inputs in this model reliable. Inclusion of costs like hospitalization costs and diagnosis costs, in addition to drug acquisition and adverse event costs, allows comprehensive representation of direct economic burden of PsA. The model results are robustly tested through both probabilistic and deterministic sensitivity analyses, while the model structure and methodology are similar to those used in a model for Canadian healthcare system [27].

This model-based analysis naturally has some limitations. One limitation of the analysis lies in the use of efficacy data from the NMA that used short-term study period of 12–16 weeks to project life-time efficacy. However, use of short term data may have underestimated the long-term efficacy of secukinumab over its comparators. For example, in one of the MAIC analysis, long term efficacy of adalimumab [25, 45], etanercept [46], and infliximab [47] were compared to that of secukinumab. In these studies covering a period of more than 3 years, secukinumab maintained its significantly higher efficacy over the TNFi. Another limitation is the assumption that efficacy for the biologics with missing data was equal to the average of other biologics. Finally, indirect costs were not included in the base case analysis as the model is from a payer’s perspective. Nevertheless, inclusion of indirect costs did not affect the model results, as explored in the alternative scenario analysis.

## Conclusion

Patients on secukinumab achieved highest gains in the quality adjusted life-years against all comparators regardless of secukinumab dose, severity of concomitant psoriasis, or prior exposure to biologics. Secukinumab was either cost-saving or cost-effective when compared with the currently used alternative treatment options for the treatment of active Psoriatic arthritis from a payer’s perspective in Finland.

## Additional file

**Additional file 1.** Sensitivity analyses and model input parameters
