# Supplementary material for: Cost-effectiveness analysis of secukinumab versus other biologics and apremilast in the treatment of active Psoriatic arthritis: a Finnish perspective
Source: Cost Eff Resour Alloc. 2018 Nov 16;16:56. doi: 10.1186/s12962-018-0162-3 (PMC6240184; doi:10.1186/s12962-018-0162-3)
Supplement: Supplementary file 1 — Additional file 1. Sensitivity analyses and model input parameters [file 12962_2018_162_MOESM1_ESM.docx]

DRAFT: Cost-effectiveness of Secukinumab in Psoriatic Arthritis in Finland

Additional Materials

**Table S1 Population Inputs**

| **Input** | **Mean** | **SD** | **Source** |
| --- | --- | --- | --- |
| Percentage male | 48.4% | N/A | Novartis (2014)^1^ |
| Age (years) | 47.96 | N/A | Novartis (2014)^1^ |
| Weight (kg) | 87.11 | 19.66 | Novartis (2014)^1^ |
| Baseline utility weight score | 0.479 | N/A | Novartis (2015c)^2^ |

N/A: Not applicable; PASI: Psoriasis Area Severity Index; SD: standard deviation

Data obtained from McInnes et al. ^3^ (FUTURE 2 trial).

**Table S2 Biologic Drug Dosing and Administration Frequency**

| **Drug** | **Dose** | **First 3 Months** | **Months 4-6** | **Subsequent**  **3-month Periods** | **Methotrexate Usage** |
| --- | --- | --- | --- | --- | --- |
| SEC | 150 mg | 7.00 | 3.00 | 3.00 | 100.0% |
| SEC | 300 mg | 7.00 | 3.00 | 3.00 | 100.0% |
| CER P | 200 mg | 10.00 | 6.00 | 6.52 | 100.0% |
| ETN/BS | 50 mg | 13.00 | 13.00 | 13.04 | 100.0% |
| ADA/BS | 40 mg | 7.00 | 6.00 | 6.52 | 100.0% |
| INF | 5 mg/kg | 3.00 | 2.00 | 1.63 | 100.0% |
| GOL | 50 mg | 3.00 | 3.00 | 3.00 | 100.0% |
| UST | 45 or 90 mg | 2.00 | 1.00 | 1.09 | 100.0% |
| APR^*^ | 30 mg | 150.00 | 184.00 | 182.63 | 100.0% |

ADA = adalimumab; APR = apremilast; BS = Biosimilar; CER P = certolizumab pegol; ETN = etanercept; GOL = golimumab; INF = infliximab; SEC = secukinumab; UST = ustekinumab.

* APR is supplied the first 14 days in a blister pack equivalent to 23 administrations of the 30mg formulation. Drug cost calculations are edited to account for these doses and costs.

Source: Finnish Medicinal Products and Prices Database (2018)^4^

**Table S3 Withdrawal Rates**

|  | Year 1 | Year 2+ |
| --- | --- | --- |
| SEC 150 | 14.00% | 7.20% |
| SEC 300 | 8.00% | 5.20% |
| CER P | 15.10% | 15.10% |
| ETN | 15.60% | 15.60% |
| ADA | 15.00% | 15.00% |
| INF | 13.90% | 8.50% |
| GOL | 29.50% | 29.50% |
| UST | 26.10% | 11.30% |
| APR | 24.10% | 24.10% |

ADA = adalimumab; APR = apremilast; ETN = etanercept; CER P = certolizumab pegol; INF = infliximab; GOL = golimumab; SEC 150 = secukinumab 150 mg; SEC 300 = secukinumab 300 mg; UST = ustekinumab.

Sources: SEC 150 Year 1 from Novartis (2015b)^5^; SEC 150 Year 2+ from Novartis (2015a)^6^; SEC 300 from Novartis (2015b)^5^; CER P from Mease et al. (2014)^7^; ETN from Sterry et al. (2010)^8^; ADA from Mease et al. (2005)^9^; INF from Torii et al. (2010)^10^; GOL from Kavanaugh et al. (2012)^11^; UST from Ritchlin et al. (2014)^12^; APR from Kavanaugh et al. (2014)^13^

**Table S4 (a) Medical and Laboratory costs: annual frequencies and costs per unit**

| **Input** | **Mean Cost (**€**)** | **Unit** | **Annual Frequency** |
| --- | --- | --- | --- |
| **Outpatient rheumatology visit** | 314.04 | Per Visit | *See Table S4 (b)* |
| **Laboratory test** |  |  |  |
| Full blood count | 2.57 | Per test | 2 |
| Erythrocyte sedimentation rate | 4.07 | Per test | 1 |
| Liver function test | 3.21 | Per test | 2 |
| Creatinine | 1.39 | Per test | 1 |

Source: National Institute for Health and Welfare. Health care costs in Finland 2011, (indexed to year 2016)^14^

Frequency for laboratory tests is based on local expert opinion

**Table S4 (b) Frequency of clinical visits^15^**

| Outpatient Days |  | SEC | CER P | ETN | ADA | INF | GOL | UST | APR |
| --- | --- | --- | --- | --- | --- | --- | --- | --- | --- |
| First 3 months |  | 1.00 | 1.00 | 1.00 | 1.00 | 3.00 | 1.00 | 1.00 | 1.00 |
| Months 4-6 |  | 1.00 | 1.00 | 1.00 | 1.00 | 2.00 | 1.00 | 1.00 | 1.00 |
| Subsequent 3-month periods | | 1.00 | 0.50 | 0.50 | 0.50 | 0.50 | 2.00 | 0.50 | 0.50 |

Frequency based on local expert opinion

**Table S5 (a) Adverse event related costs**

| **Adverse event** | **Mean Cost (**€) | **Source** |
| --- | --- | --- |
| Tuberculosis | 14,445.00* | ^14^ |
| Other serious infection | 3,013.10 | ^14^ |
| Malignancy | 24,832.00 | ^16^ |

Source: National Institute for Health and Welfare. Health care costs in Finland 2011, (indexed to year 2016)

*Care consisting of 14 days hospital care and monthly 1 year follow up in specialized health care unit.

**Table S5 (b) Adverse event rates**

| **Drug** | **Infection** | **Malignancy** |
| --- | --- | --- |
| SEC 150 mg^17^ | 0.63% | 0.13% |
| SEC 300 mg^17^ | 0.63% | 0.13% |
| Certolizumab Pegol^7^ | 0.79% | 0.39% |
| Etanercept^8^ | 0.44% | 0.15% |
| Adalimumab^18^ | 0.70% | 0.12% |
| Infliximab^10^ | 0.48% | 0.48% |
| Golimumab^11^ | 0.34% | 0.17% |
| Ustekinumab^12^ | 0.21% | 0.21% |
| Apremilast^13^ | 0.65% | 0.32% |
| Standard of Care | 0.00% | 0.00% |

**Table S5 (c) Indirect Costs based on HAQ scores**

| **HAQ Score** | | **% employed** | **Cost per 3 months*** |
| --- | --- | --- | --- |
| **min** | **max** |  |  |
| 0.0 | 0.6 | 76% | €10,509.50 |
| 0.6 | 1.1 | 59% |  |
| 1.1 | 1.6 | 55% |  |
| 1.6 | 2.1 | 56% |  |
| 2.1 | 2.6 | 32% |  |
| 2.6 | 3.0 | 13% |  |

*Based on the annual cost of loss of productivity €42,038.

Source: National Institute for Health and Welfare. Health care costs in Finland 2011, (indexed to year 2016)^14^; (Conaghan 2016)^19^;

**Table S6 Utility weight Inputs**

| **Parameter** | **FUTURE 2 (Base Case)** | **York Model (Scenario)** |
| --- | --- | --- |
| Intercept | 0.839 | 0.897 |
| HAQ disability index score | −0.172 | -0.298 |
| PASI total score | −0.002 | -0.004 |
| Baseline EQ-5D | 0.210 | N/A |
| TNF-α status ^a^ |  | N/A |
| Inadequate responder | -0.008 | N/A |
| Sex ^b^ |  |  |
| Male | -0.019 | N/A |
| Age (years) | -0.003 | N/A |

HAQ = Health Assessment Questionnaire; N/A = not applicable; PASI = Psoriasis Area Severity Index; TNF-α = tumor necrosis factor alpha.

a Naïve is the reference for TNF-α status.

b Female is the reference for sex.

Sources: York values from Rodgers et al. (2011)^20^; FUTURE 2 values from a mixed parameters modeled using trial data (not published).

**Table S7 Mortality Inputs**

| **Input** | **Relative Risk** |  |
| --- | --- | --- |
| **Disease-specific mortality** | | |
| Male | 1.65^21^ | |
| Female | 1.59^21^ | |
| **Adverse event mortality** | | |
| Infection | 1.65^22^ | |
| Malignancy–Year 1 | 1.65^22^ | |
| Malignancy–Year 2 | 1.41^22^ | |
| Malignancy–Year 3 | 1.41^22^ | |
| Malignancy–Year 4 | 1.41^22^ | |
| Malignancy–Year 5 | 1.41^22^ | |

**Table S8 Scenario Analysis**

| Base Case Results | | | Dominates all biologics, APR and biosimilars except INF. But INF is not cost-effective vs SEC 150 | Cost-effective vs all biologics, APR & dominates INF, but not cost effective against ADA and ETN biosimilar | Cost-effective vs all biologics, APR except the biosimilars |
| --- | --- | --- | --- | --- | --- |
| Parameters | Base Case assumptions | Alternate assumptions | Naïve: non moderate to severe psoriasis: 150 mg | Naïve: moderate to severe psoriasis: 300 mg | Experienced: 300 mg |
| Time horizon | 60 years | 5 years & 10 years | Similar result for 5 & 10 years | At 5 years, SEC was only cost effective against UST.  At 10 years, SEC was cost effective against CER P, ADA, GOL & UST. | At 5-years, SEC had higher QALYs against all comparators and was cost-effective vs UST, and dominated INF.  At 10 years, SEC had higher QALYs against all comparators and was cost effective against INF and UST. |
| Discounting | 3% | 0% (both cost and QALYs) | SEC dominated all biologics and was cost effective against APR | SEC was cost-effective vs all biologics, APR except ETN BS and ADA BS. | SEC was cost effective against all biologics, APR and ADA biosimilar, except ETN BS. |
|  |  | 5% (both cost and QALYs) | Similar to base case | Similar to base case | SEC was cost-effective vs all biologics except ETN, ADA BS and APR. |
| Indirect Costs | Not included | Included | Similar to base case | SEC was cost effective against all biologic APR, ETN biosimilar & ADA biosimilar | SEC was cost effective against all biologics, APR and biosimilars and dominates INF. |
| HAQ rebound assumption | Return to baseline | Initial gain retained | Similar to base case | SEC was cost effective against all comparators except ETN, ETN BS, ADA BS and APR. | SEC had higher QALYs compared to all biologics & APR. SEC was cost-effective vs INF and UST. |
| Efficacy | PsARC | PASI ≥75 | SEC dominated all biologics & APR. | SEC was cost effective against all biologics and APR except ETN BS and ADA BS. | SEC was cost-effective against all biologics, except ETN, ADA BS and APR. |
|  |  | PsARC  &  PASI ≥75 | Similar to base case | Similar to base case | Similar to base case |
| Utility | FUTURE 2 trial | York model utilities | Similar to base case | SEC dominated INF and was cost effective against UST.  SEC had higher QALYs over other biologics and APR. | SEC had higher QALYs against all biologics. |
| Disutilities | Not included | Included | Similar to Base case | Similar to Base case | Similar to Base case |
| Adverse events | Included | Not included | Similar to base case | SEC was cost effective against all biologics and APR, except INF, ETN BS, ADA BS. | Similar to base case |
| Disease related costs | Included | Not included | Similar to base case | Similar to base case | Similar to base case |

**Table S9 Probabilistic Sensitivity Analysis**

| Outcome | SEC | CER P | ETN biosimilar | ADA biosimilar | INF | GOL | UST | APR |
| --- | --- | --- | --- | --- | --- | --- | --- | --- |
| SEC 150 mg in Biologic-Naïve PsA without moderate to severe PsO | | | | | | | | |
| **Mean Total Cost(€)** | 180,730 | 195,239 | 185,757 | 185,411 | 222,890 | 188,314 | 203,580 | 183,305 |
| SD | 11,126 | 14,952 | 10,672 | 10,903 | 14,506 | 11,711 | 18,133 | 12,383 |
| **Mean Total QALYs** | 8.07 | 7.45 | 7.84 | 7.63 | 8.19 | 7.31 | 7.61 | 7.26 |
| SD | 1.02 | 0.85 | 0.8 | 0.81 | 0.76 | 0.86 | 0.87 | 0.87 |
| **Mean NMB (€)** | 62,723 | 29,698 | 49,838 | 43,989 | 23,245 | 32,056 | 26,042 | 36,467 |
| SD | 31,058 | 28,190 | 26,940 | 27,431 | 25,740 | 28,927 | 28,042 | 28,938 |
| **Max NMB*** | 70.67% | 0.00% | 26.67% | 2.66% | 0.20% | 0.00% | 0.00% | 0.00% |
| SEC 300 mg in Biologic-Naïve PsA with moderate to severe PsO | | | | | | | | |
| **Mean Total Cost(€)** | 223,198 | 199,734 | 190,833 | 191,048 | 227,957 | 193,987 | 207,101 | 188,459 |
| SD | 29,834 | 14,922 | 10,980 | 11,110 | 13,677 | 11,829 | 17,485 | 12,611 |
| **Mean Total QALYs** | 7.96 | 6.88 | 7.26 | 7.07 | 7.64 | 6.75 | 7.05 | 6.69 |
| SD | 1.29 | 0.89 | 0.82 | 0.83 | 0.81 | 0.87 | 0.87 | 0.89 |
| **Mean NMB (€)** | 56,946 | 8,197 | 28,579 | 22,835 | 2,429 | 10,018 | 4,759 | 14,279 |
| SD | 41,060 | 28,337 | 27,514 | 27,444 | 26,258 | 29,465 | 27,763 | 29,406 |
| **Max NMB*** | 83.70% | 2.00% | 8.15% | 0.00% | 2.00% | 0.00% | 0.00% | 4.15% |
| SEC 300 mg in Biologic-Experienced population | | | | | | | | |
| **Mean Total Cost(€)** | 247,695 | 203,232 | 193,320 | 190,936 | 224,256 | 192,674 | 209,365 | 187,704 |
| SD | 13,628 | 10.346 | 9,831 | 10,146 | 10,907 | 10,182 | 10,025 | 10,183 |
| **Mean Total QALYs** | 8.82 | 7.22 | 7.57 | 7.28 | 7.82 | 6.99 | 7.33 | 6.93 |
| SD | 0.69 | 0.8 | 0.78 | 0.8 | 0.76 | 0.85 | 0.78 | 0.85 |
| **Mean NMB (€)** | 68,887 | 16,202 | 36,729 | 30,642 | 12,402 | 20,403 | 13,860 | 23,400 |
| SD | 21,127 | 25,456 | 24,756 | 25,503 | 24,164 | 27,273 | 24,839 | 27,331 |
| **Max NMB*** | 89.67% | 0.00% | 8.33% | 0.00% | 2.00% | 0.00% | 0.00% | 0.00% |

ADA = adalimumab; APR = apremilast; CER P = certolizumab pegol; ETN = etanercept; GOL = golimumab; INF = infliximab; NMB = net monetary benefit; QALY = quality-adjusted life-year; SEC = secukinumab; SD = standard deviation; SoC = standard of care; UST = ustekinumab.

*Maximum NMB calculated at WTP threshold of €30,000.

**Figure S1** Network of RCTs measuring PsARC outcomes in patients with Psoriatic Arthritis^23^


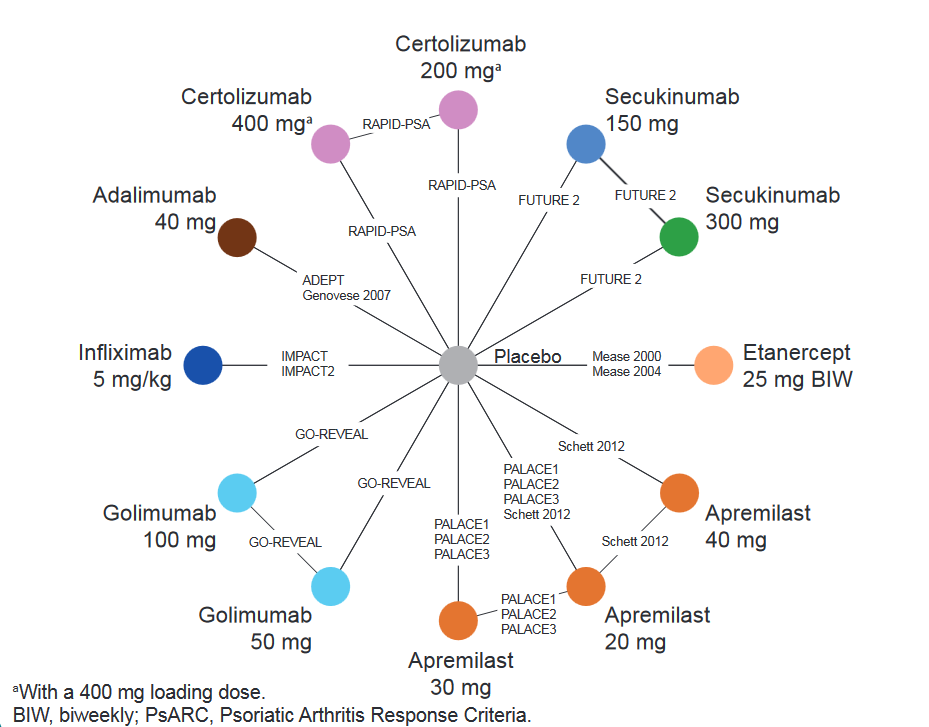


**Figure S2** Predicted proportion of PASI75 and 90 responders at 12-16 weeks in the mixed population as per Network Meta-Analysis^23^

**
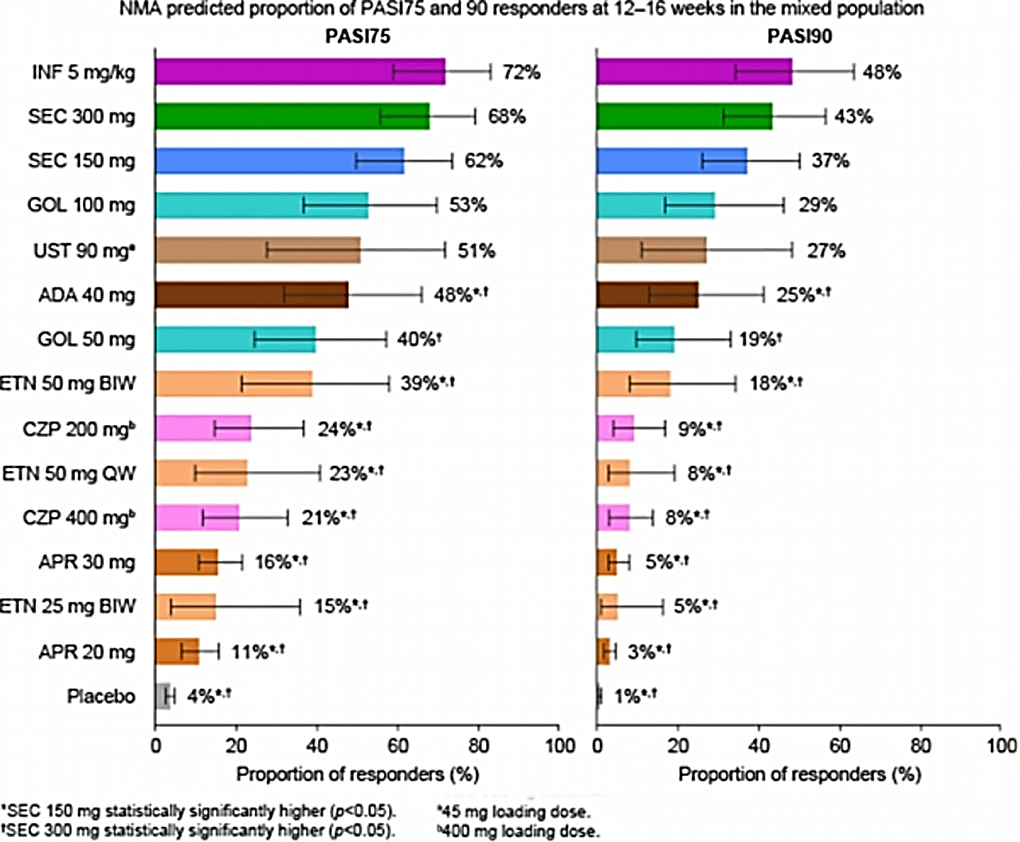
**

PsARC and PASI scores assessment based on 20 Randomized Controlled Trial (RCTs) with 6021 active PsA patients. Total six trials with PsARC outcome and 12 trials included 3339 biologic-naïve patients.

**Figure S3** One-Way Deterministic Sensitivity Analysis: Tornado Diagrams for SEC 150 mg in Biologic-Naïve PsA without moderate to severe PsO

*The NMB has been calculated considering WTP threshold of €30,000*


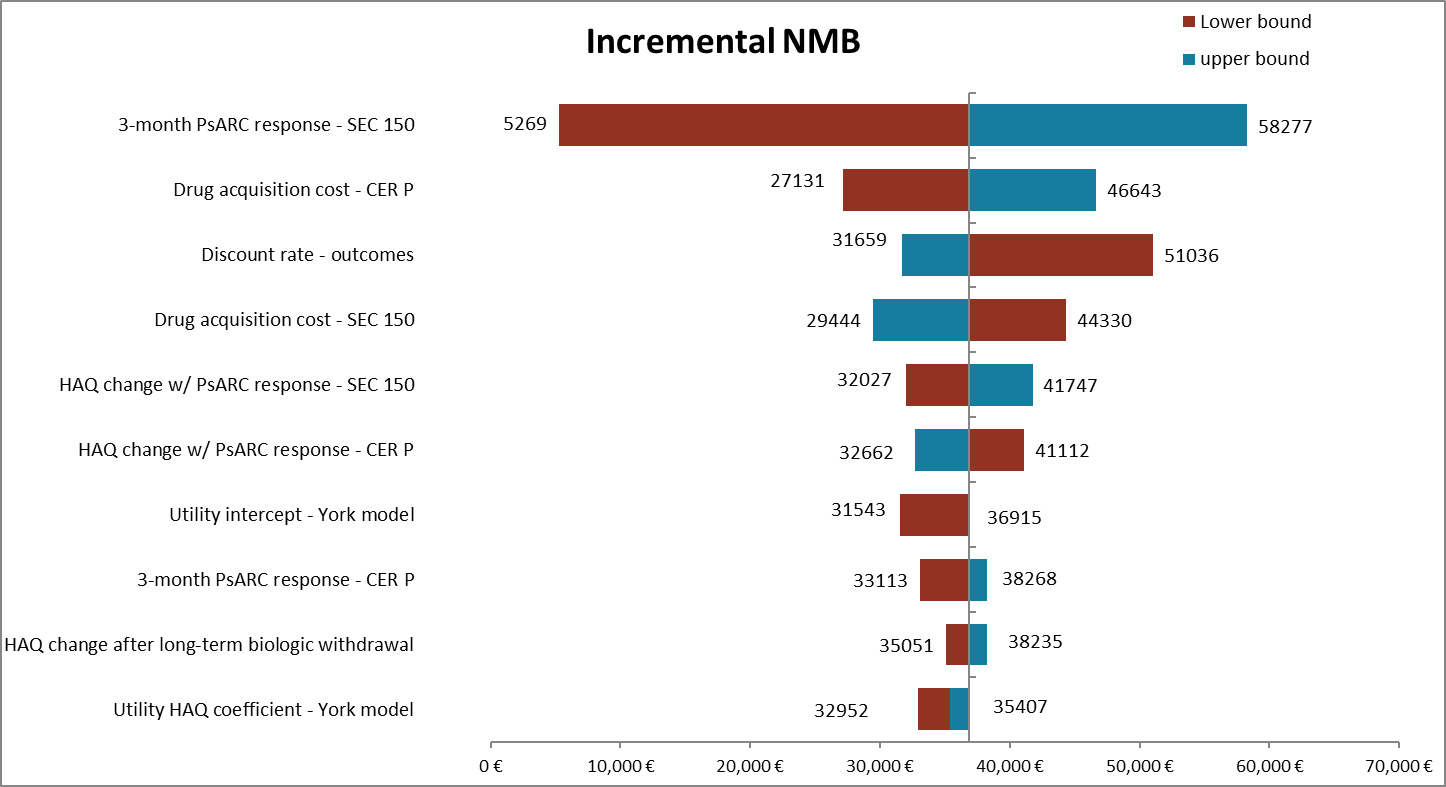


Vs CER P


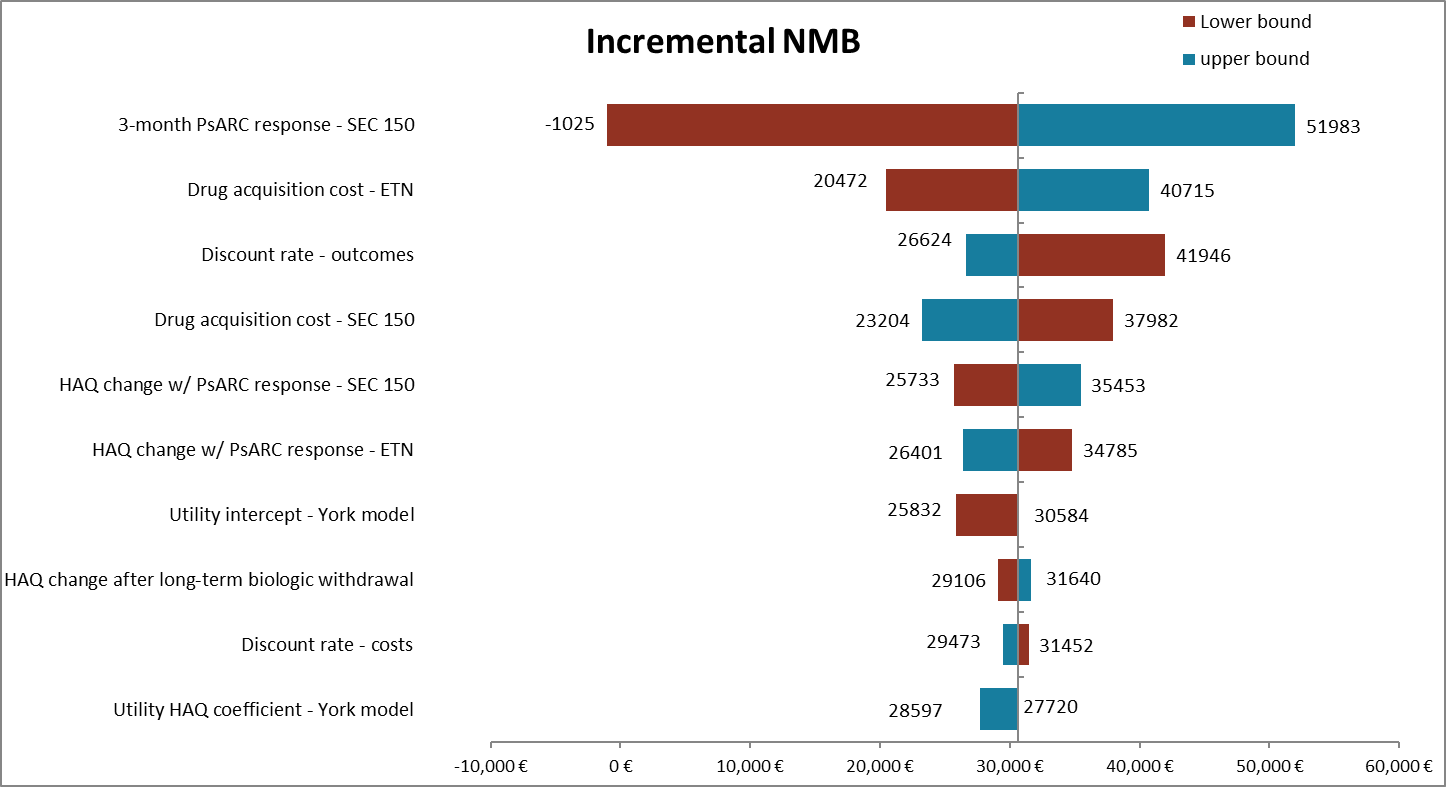


Vs ETN


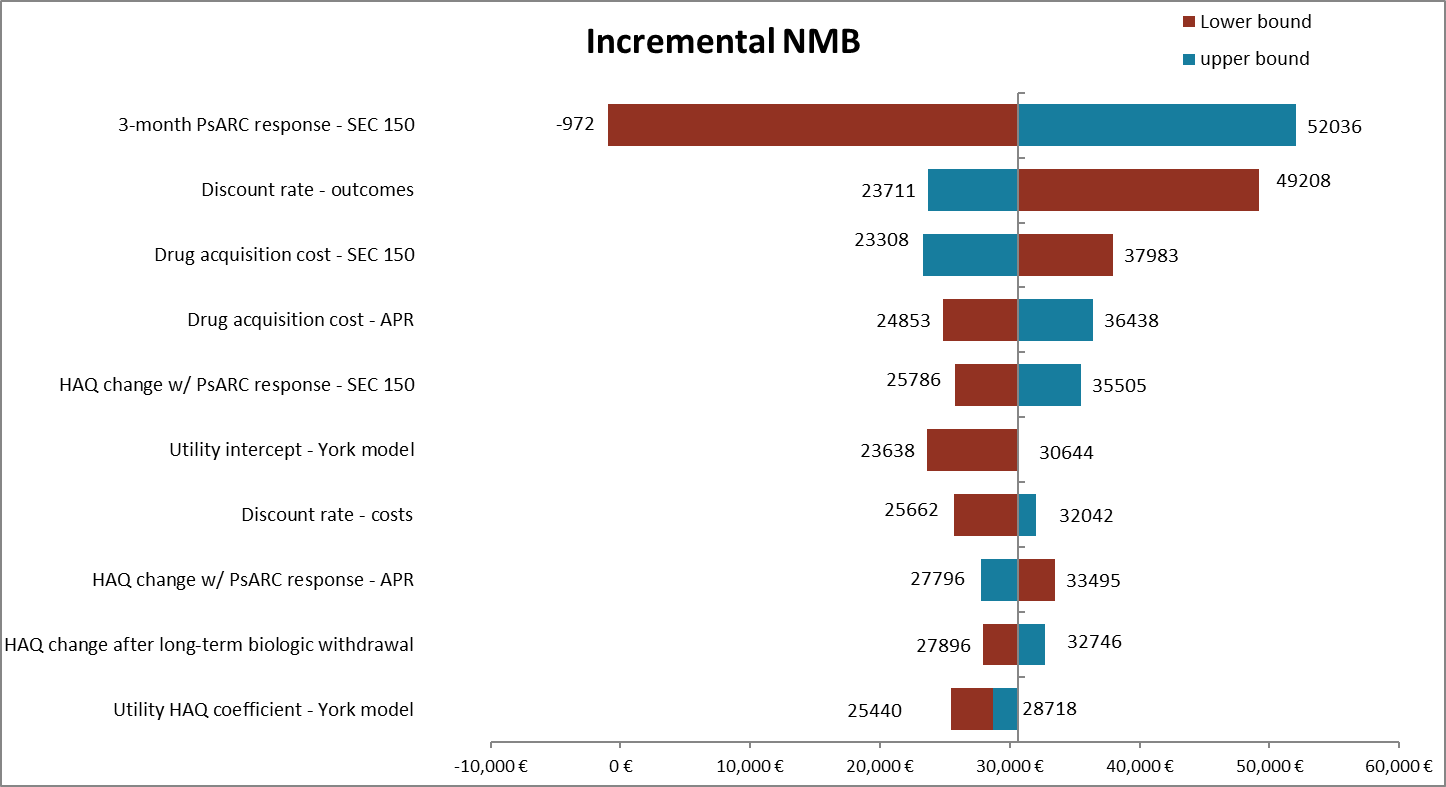


Vs APR


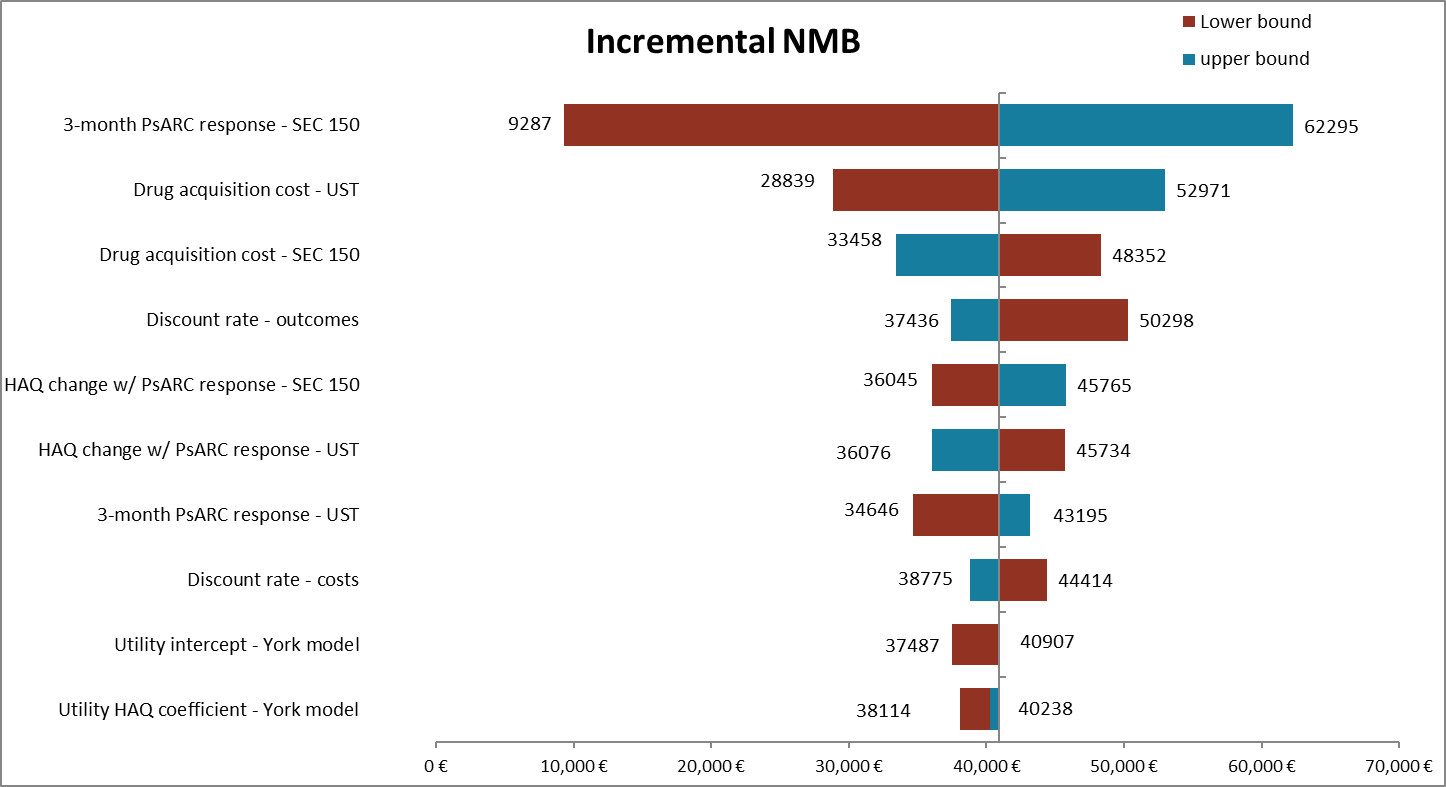


Vs UST


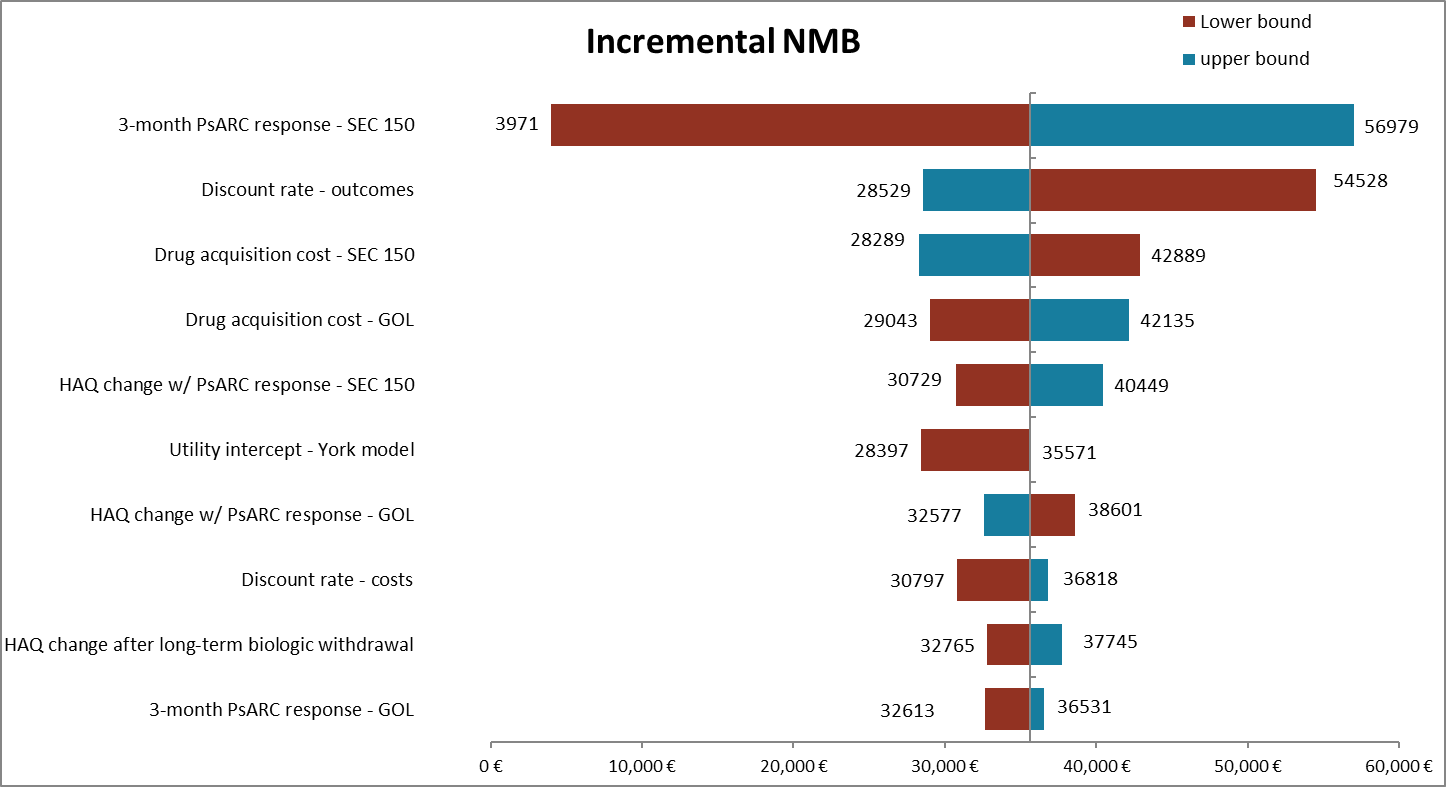


Vs GOL


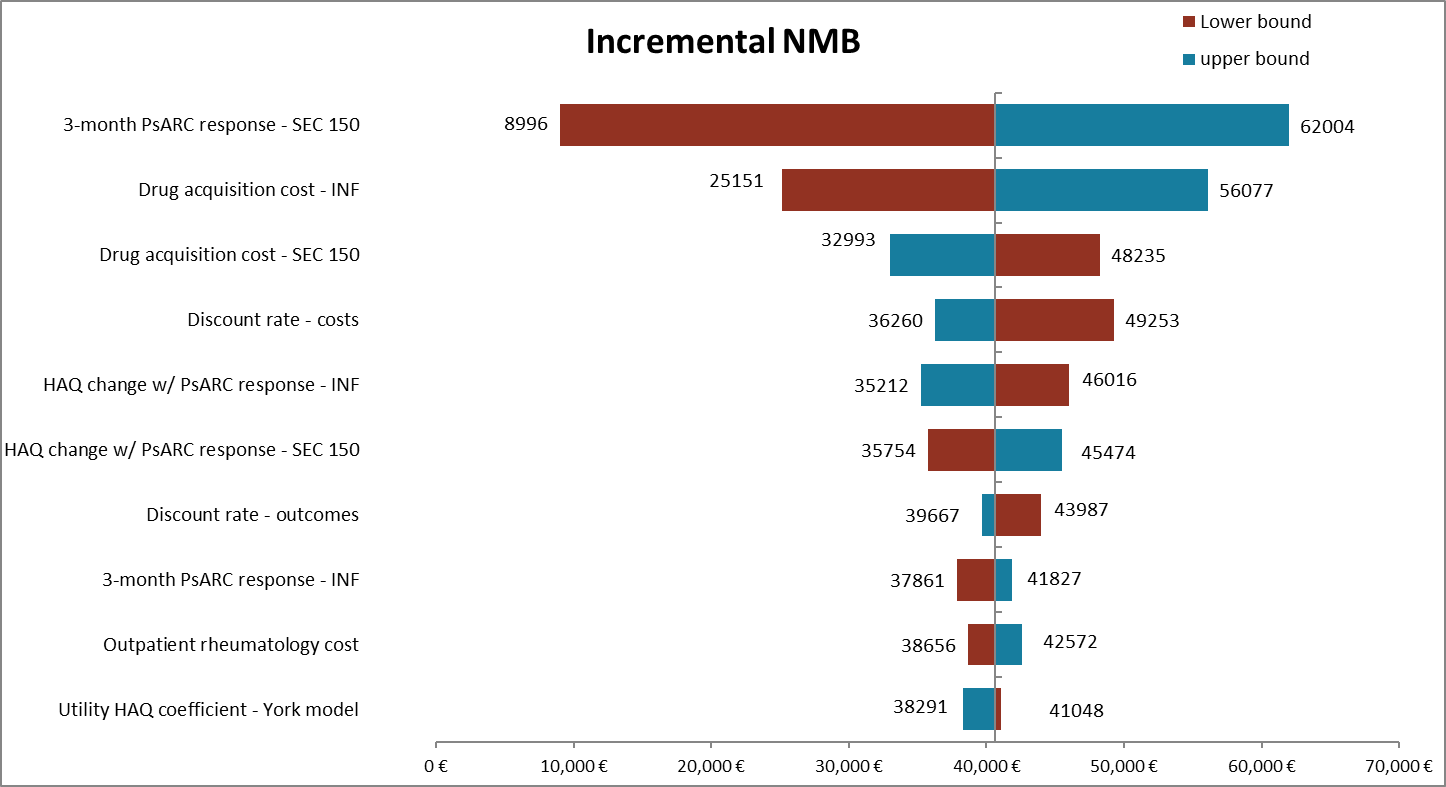


Vs INF


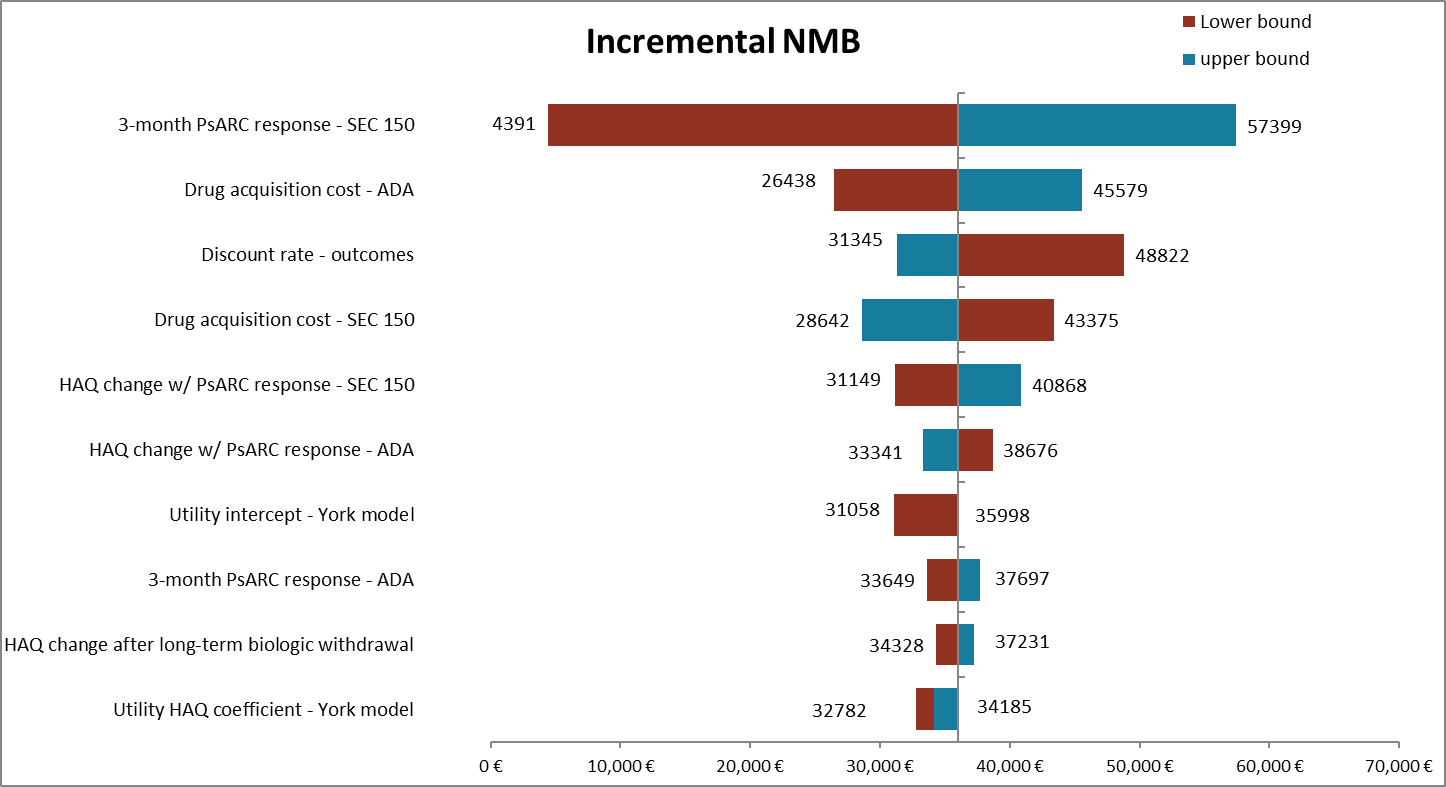


Vs ADA

**Figure S4** One-Way Deterministic Sensitivity Analysis: Tornado Diagrams for SEC 300 mg in Biologic-Naïve PsA with moderate to severe PsO

*The NMB has been calculated considering WTP threshold of €30,000*


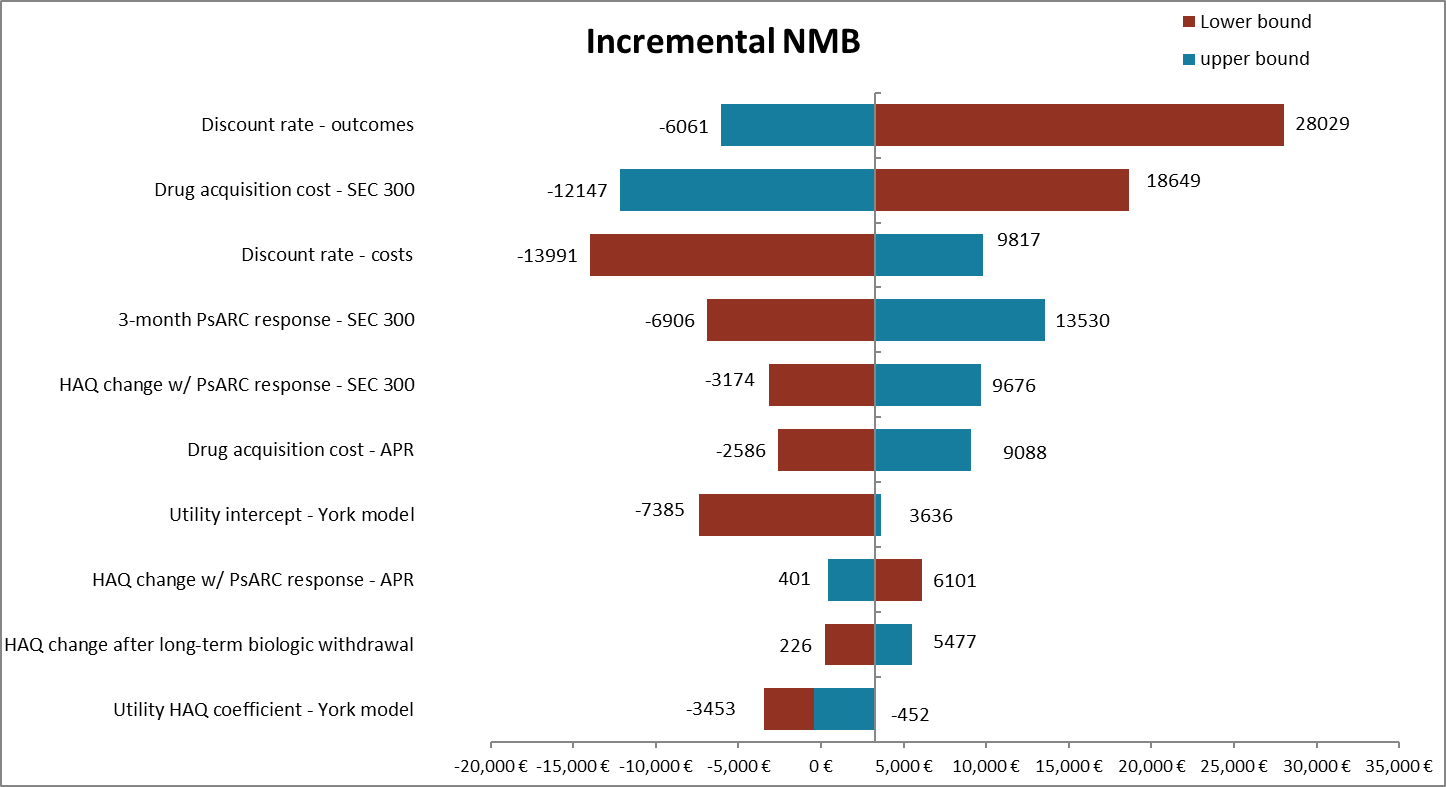

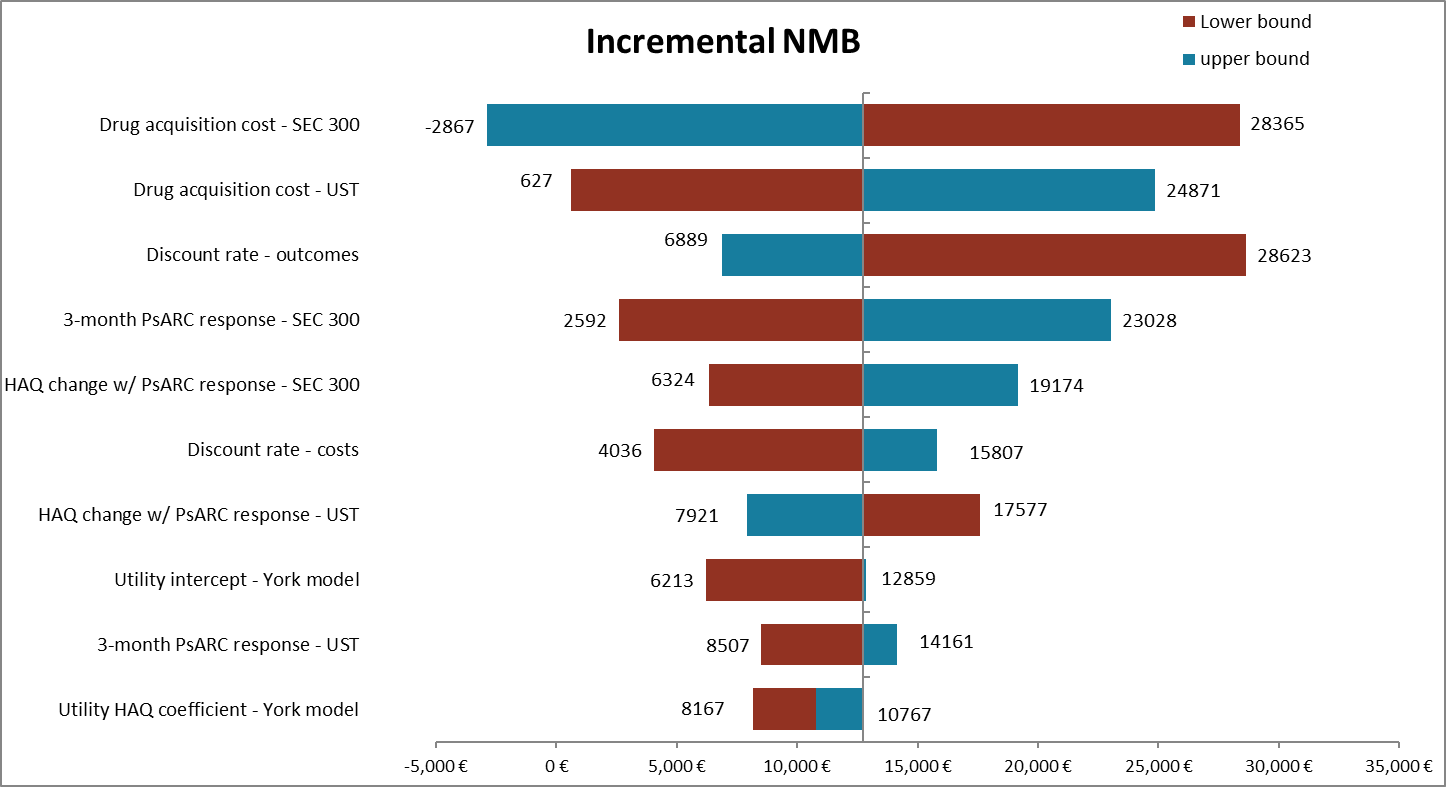


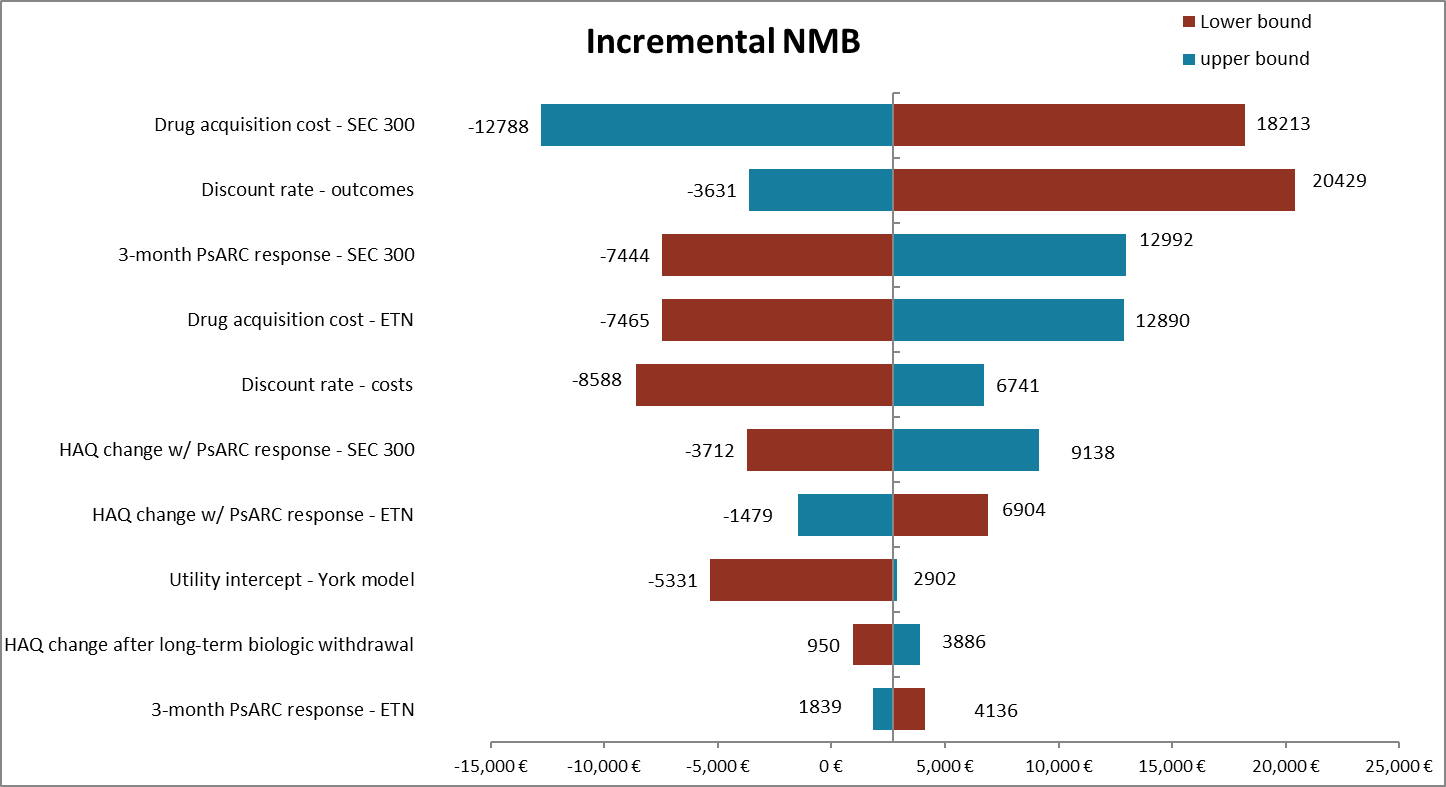


Vs ETN


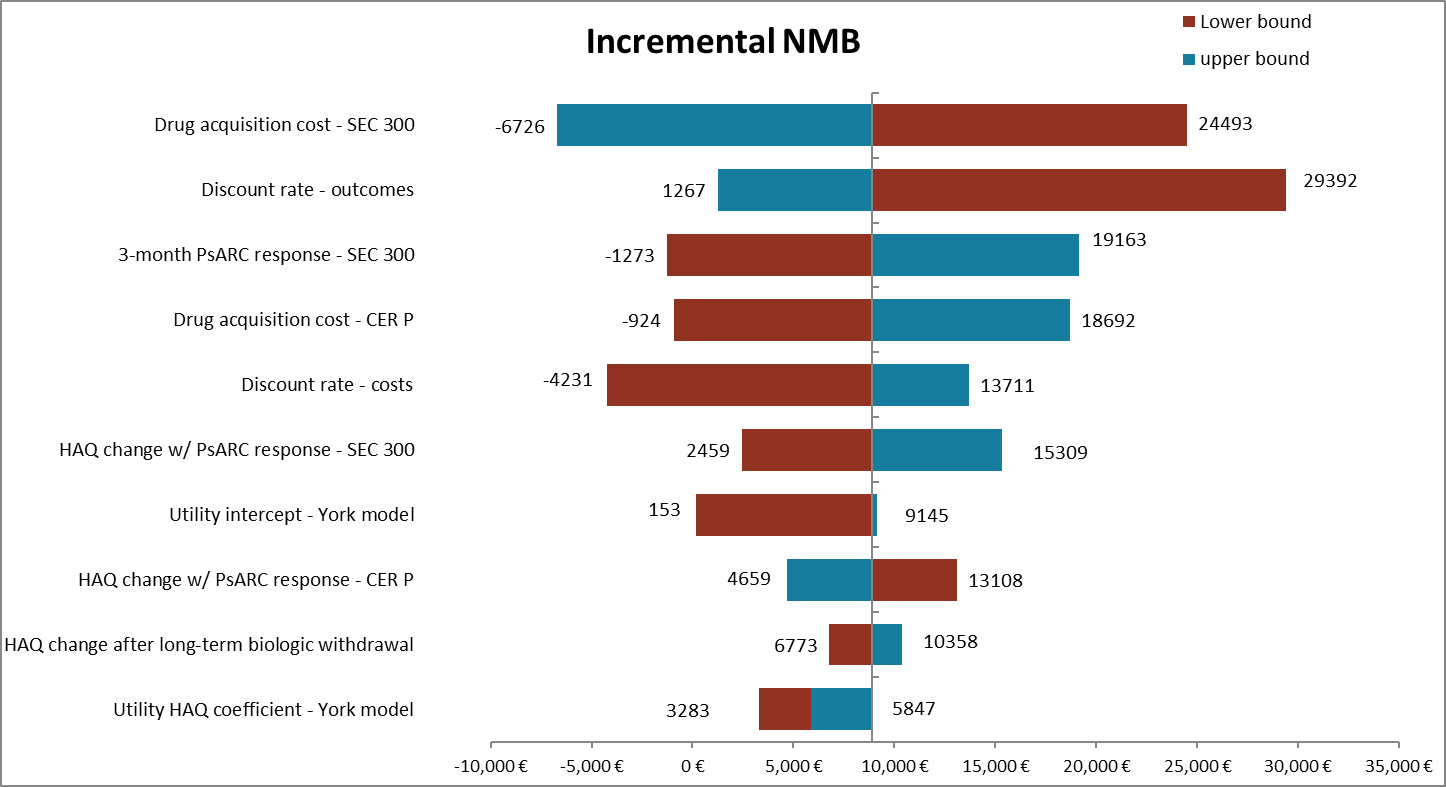


Vs CER P

Vs APR

Vs UST


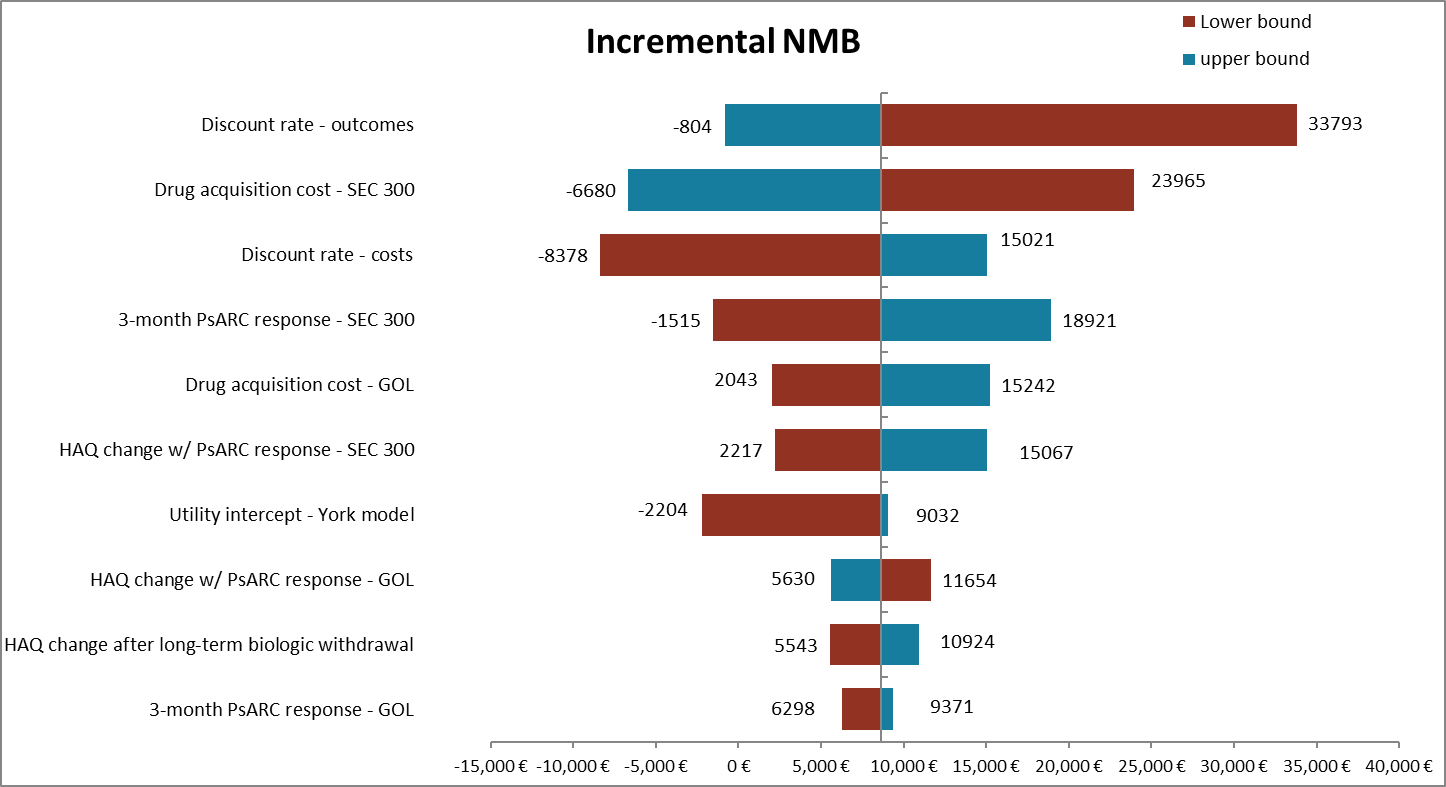


Vs GOL


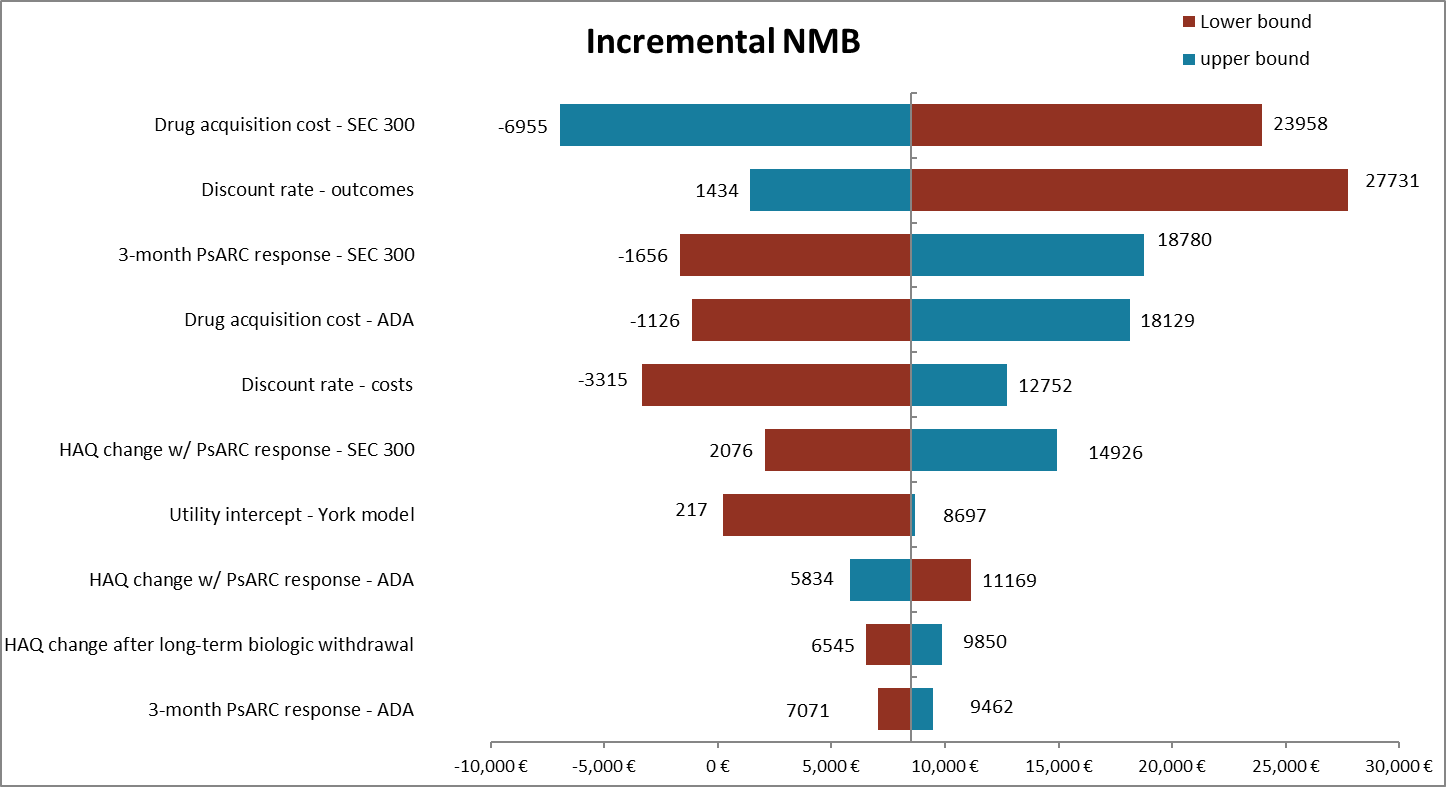


Vs ADA


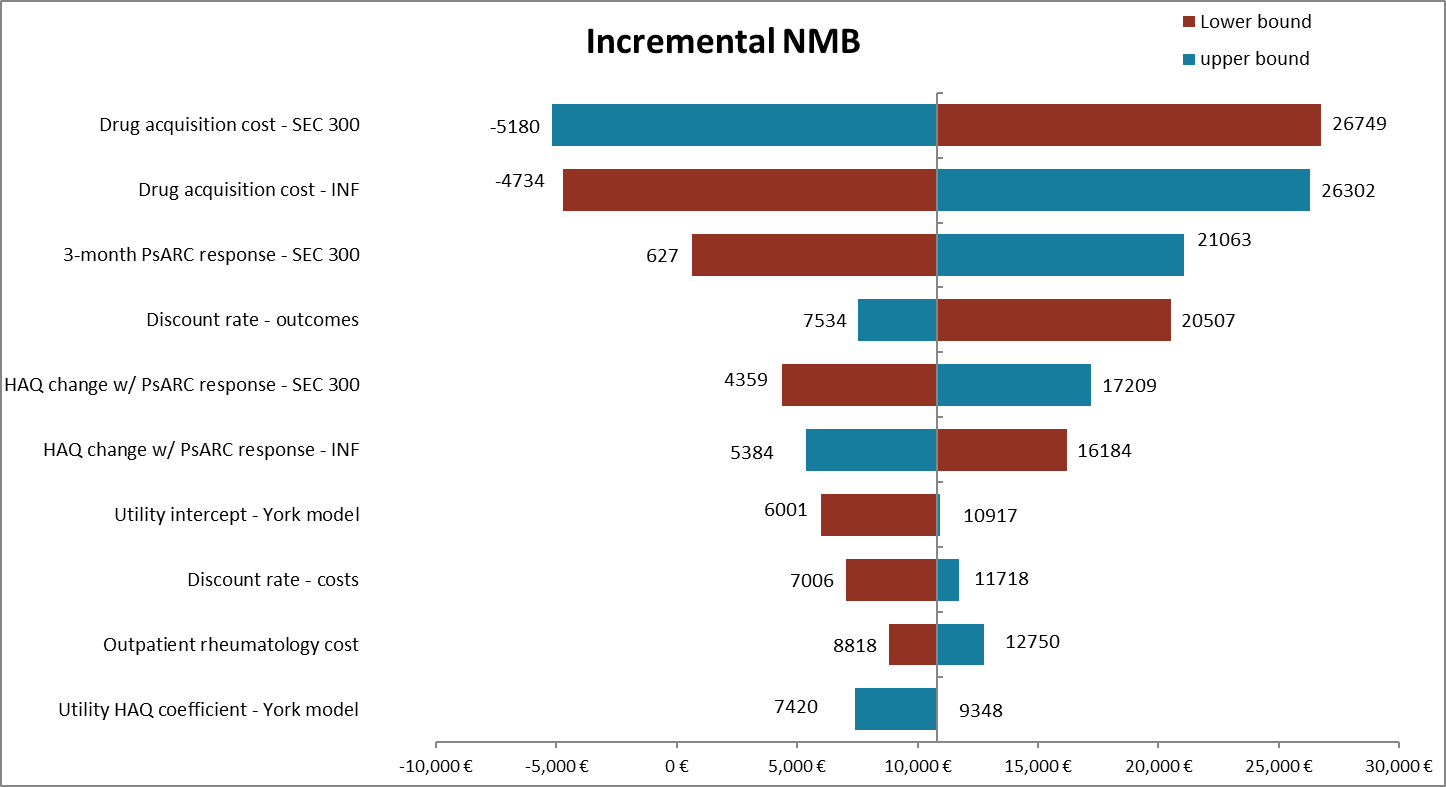


Vs INF

**Figure S5** One-Way Deterministic Sensitivity Analysis: Tornado Diagrams for SEC 300 mg in Biologic-Experienced Population

*The NMB has been calculated considering WTP threshold of €30,000*


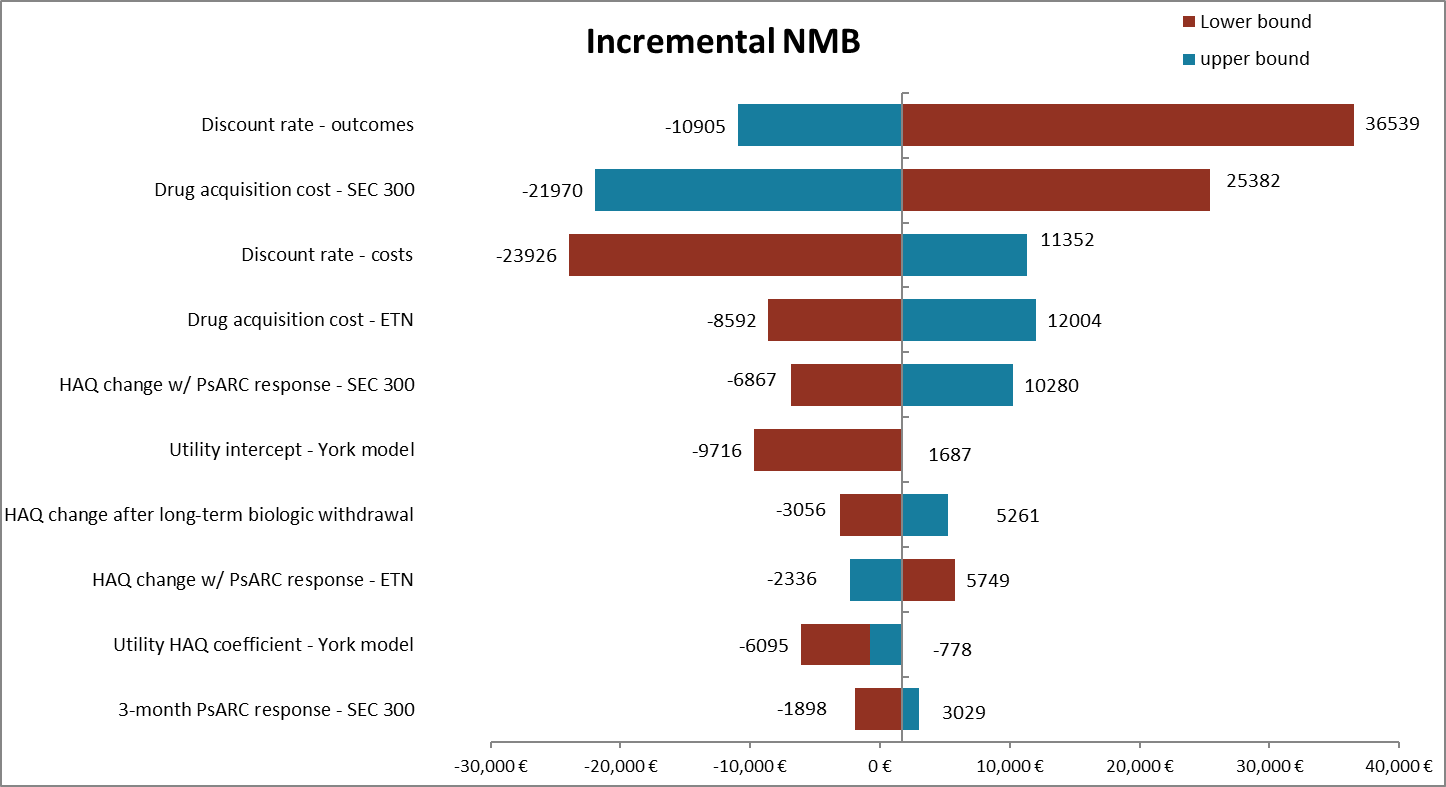


Vs ETN


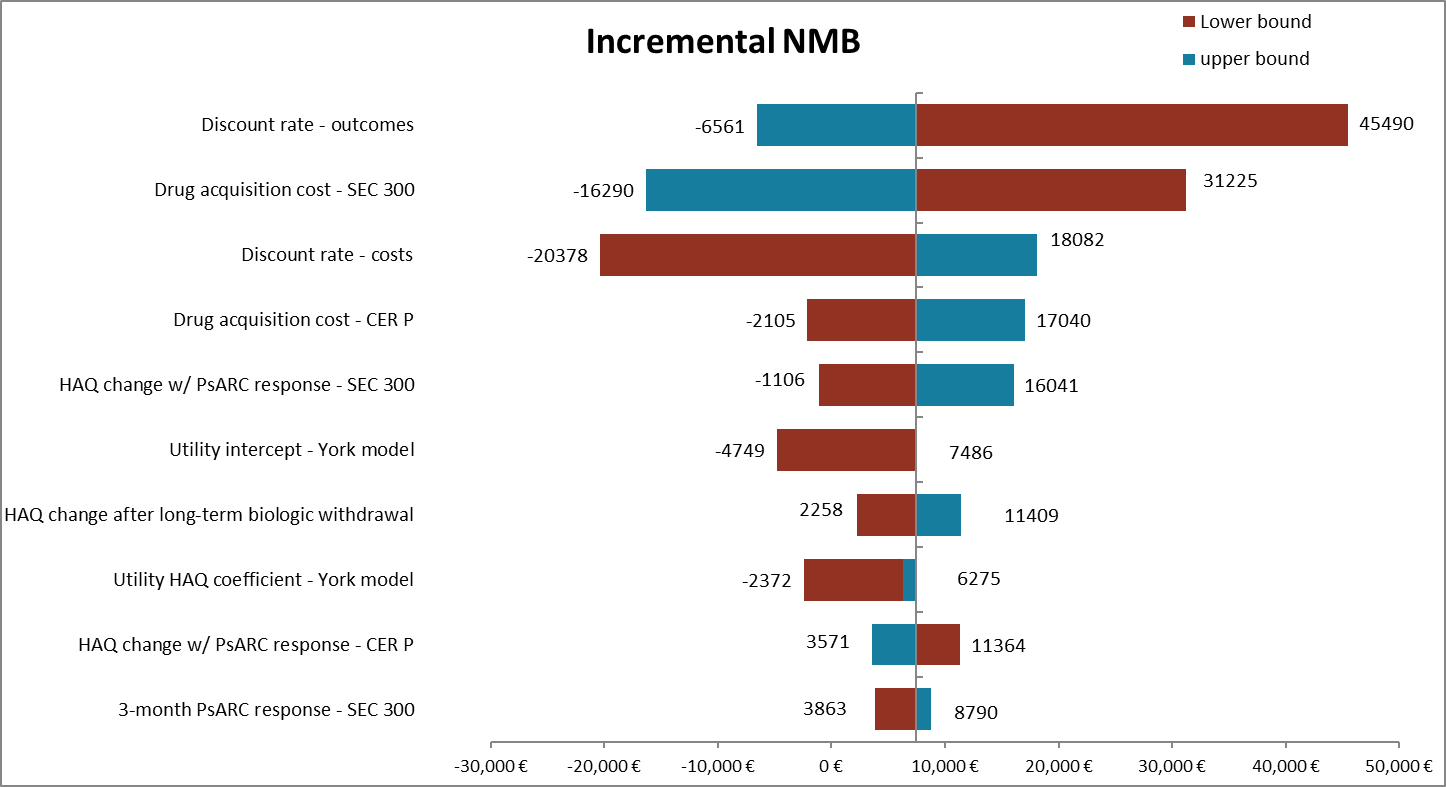


Vs CER P


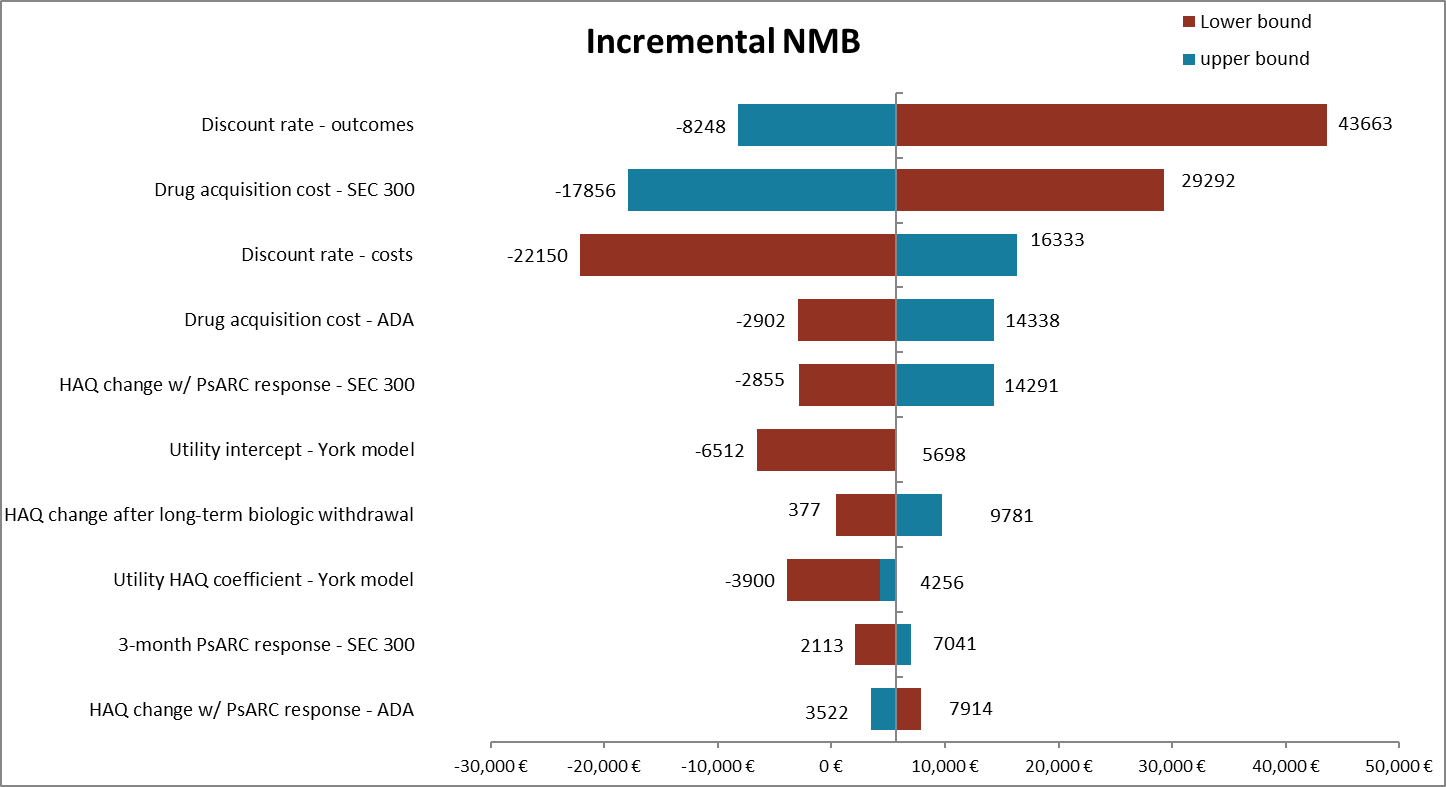


Vs ADA


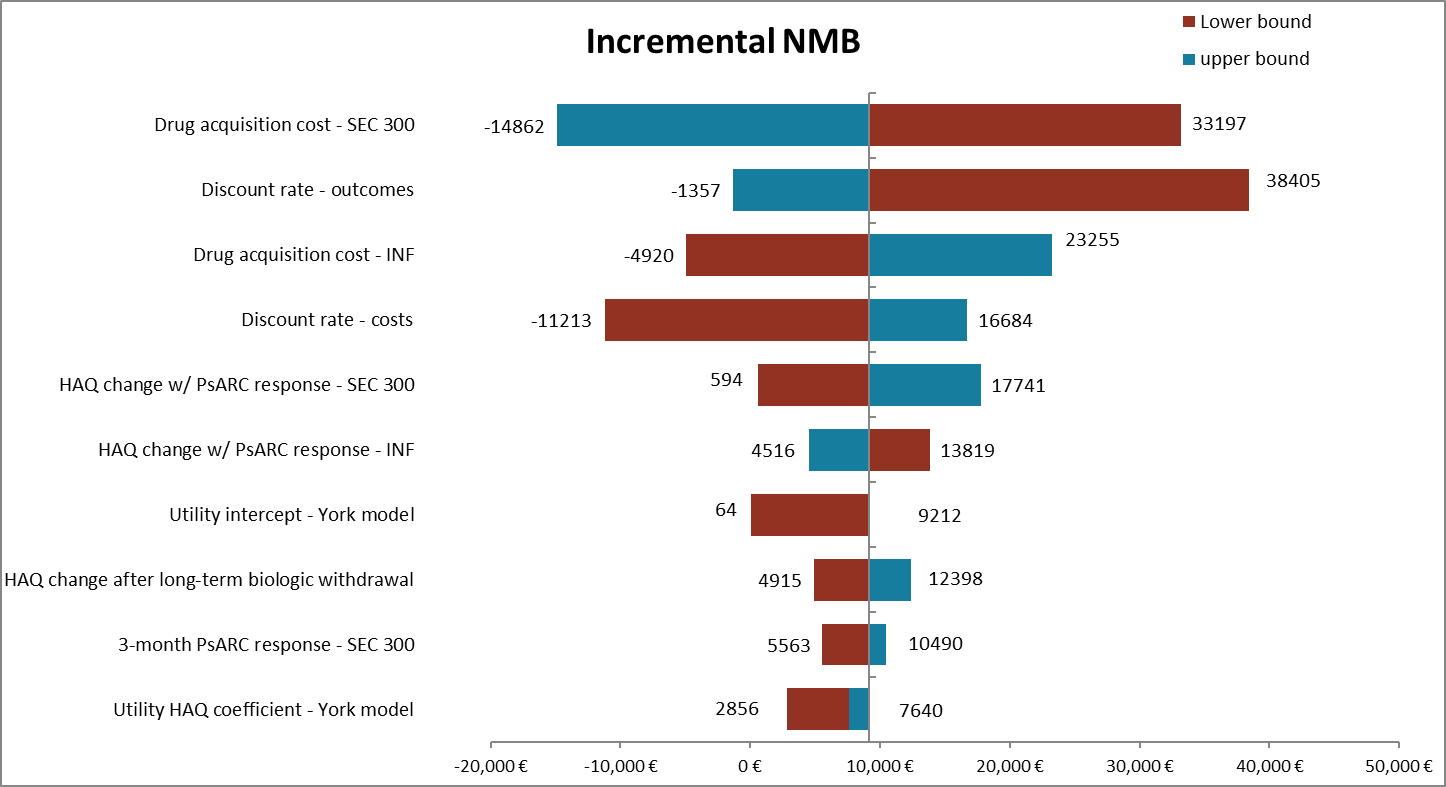


Vs INF


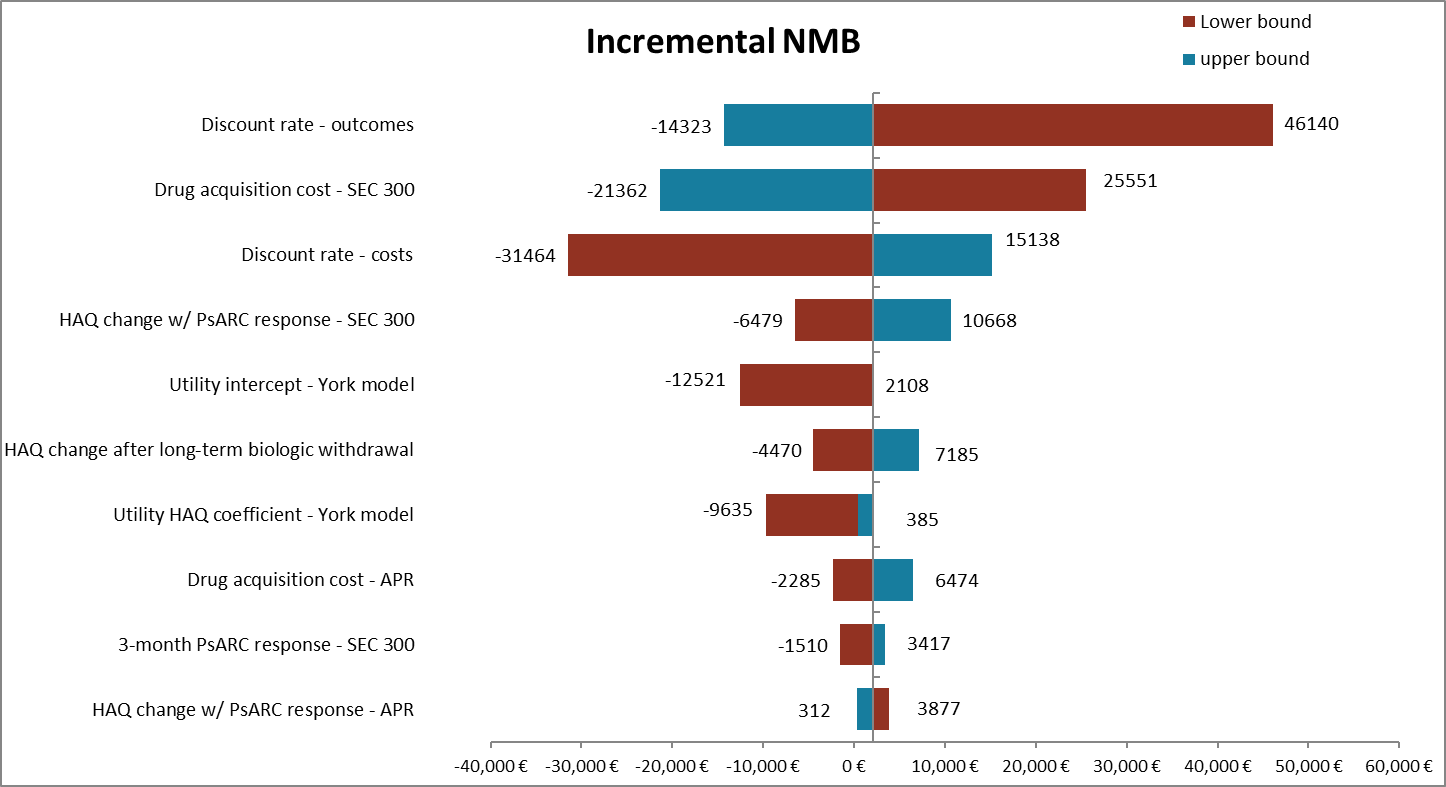


Vs APR


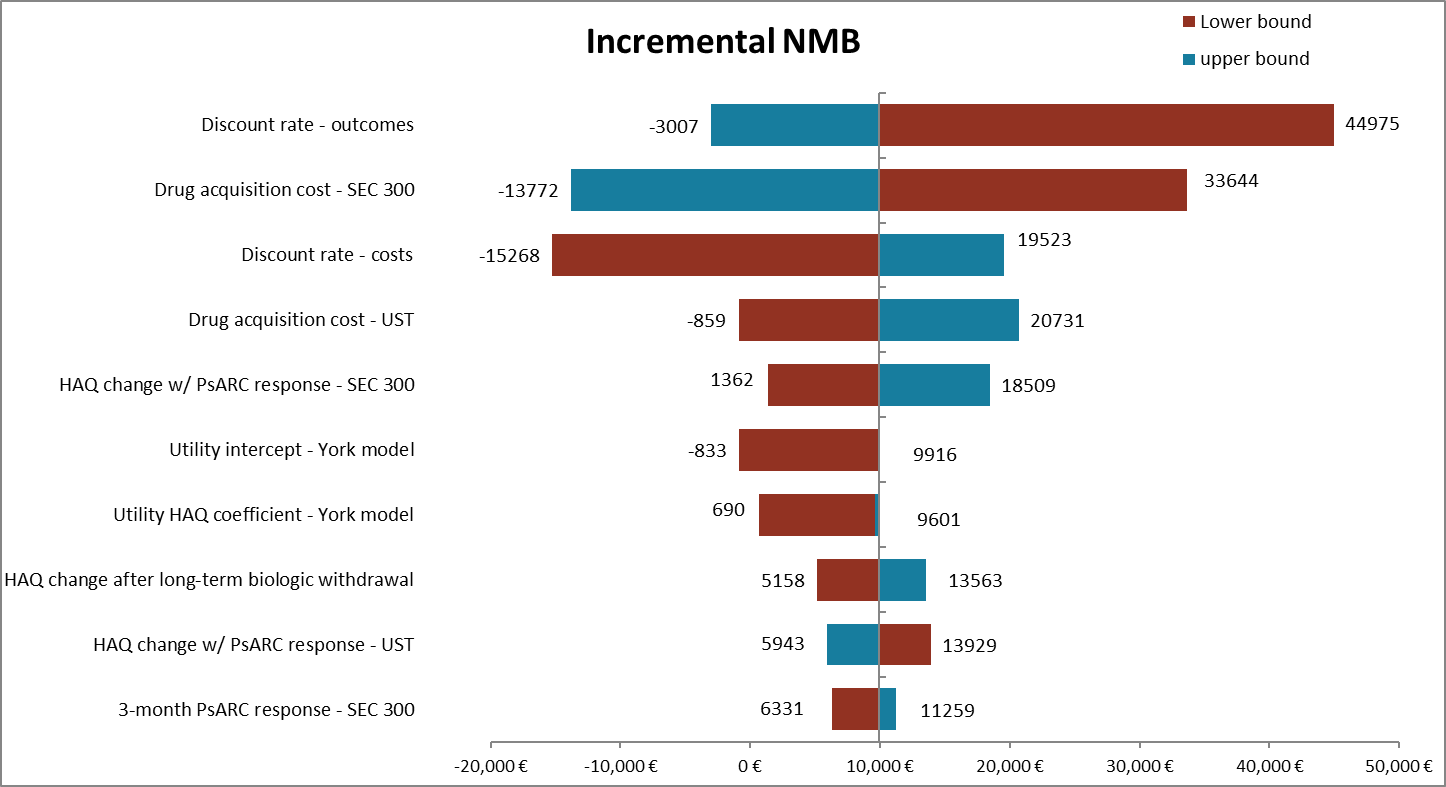


Vs UST


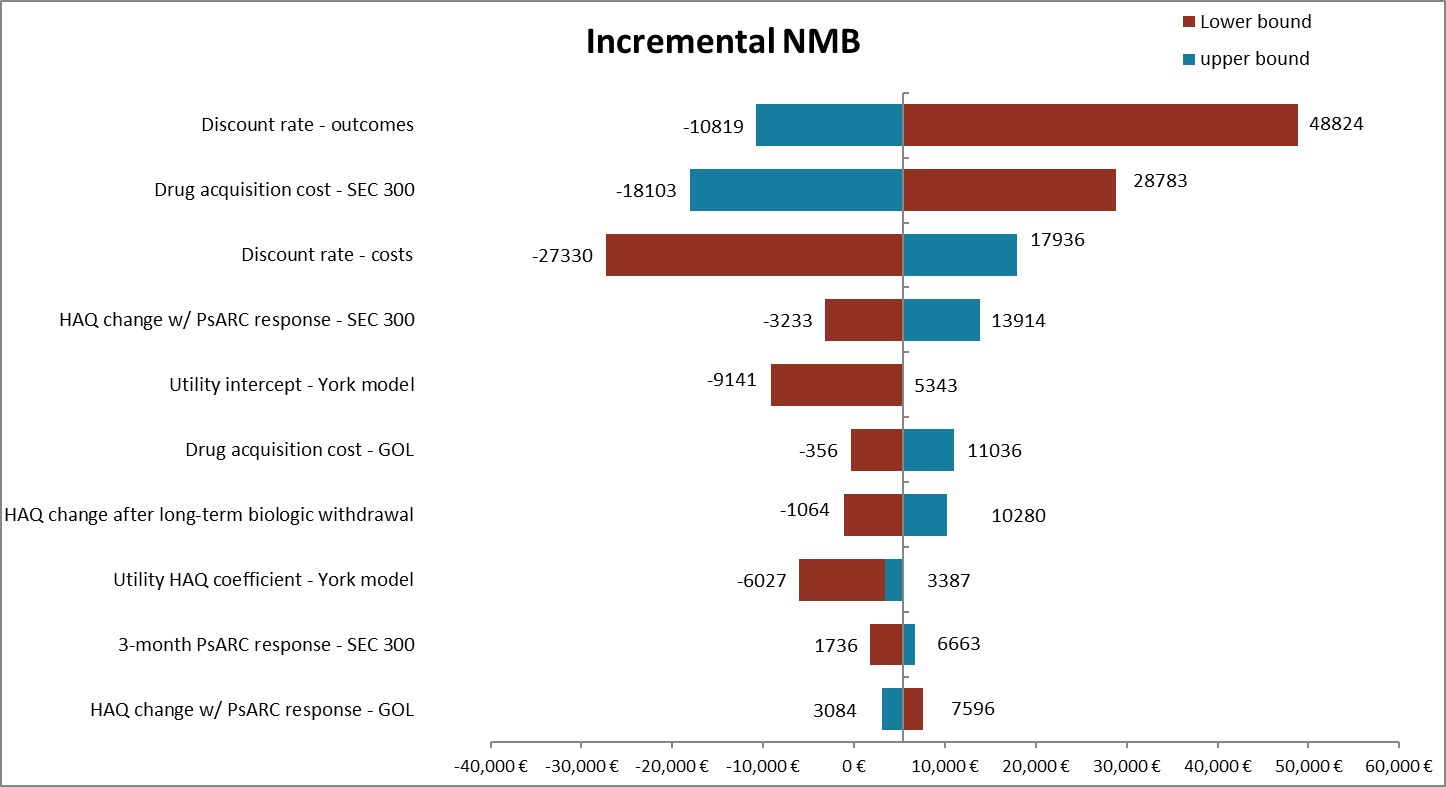


Vs GOL

**References**

1. Novartis. A Phase III randomized, double-blind, placebo-controlled multicenter study of subcutaneous secukinumab in prefilled syringes to demonstrate the efficacy at 24 weeks and to assess the long term efficacy, safety and tolerability up to 5 years in patients wi. Protocol number CAIN457F2312, p. Data on file.

2. Novartis c. FUTURE 2 study, s.l.: Data on File.

3. McInnes IB, Mease PJ, Kirkham B, et al. Secukinumab, a human anti-interleukin-17A monoclonal antibody, in patients with psoriatic arthritis (FUTURE 2): a randomised, double-blind, placebo-controlled, phase 3 trial. *Lancet*. 2015;386(9999):1137-46.

4. Finnish Medicinal Products and Prices Database. 2018.

5. Novartis b. A phase III randomized, double-blind, placebo-controlled multicenter study of subcutaneous secukinumab in prefilled syringes to demonstrate the efficacy at 24 weeks and to assess the long term efficacy, safety and tolerability up to 5 years in patients wi. Protocol Number CAIN457F2312, p. Data on File.

6. Novartis a. A randomized, double-blind, placebo-controlled, multicenter study of secukinumab to demonstrate the efficacy at 24 weeks and to assess the long term safety, tolerability and efficacy up to 2 years in patients with active psoriatic arthritis. Protocol number CAIN457F2306, p. Data on File.

7. Mease PJ, Fleischmann R, Deodhar AA, et al. Effect of certolizumab pegol on signs and symptoms in patients with psoriatic arthritis: 24-week results of a Phase 3 double-blind randomised placebo-controlled study (RAPID-PsA). *Ann Rheum Dis*. 2014;73(1):48-55.

8. Sterry W, Ortonne JP, Kirkham B, et al. Comparison of two etanercept regimens for treatment of psoriasis and psoriatic arthritis: PRESTA randomised double blind multicentre trial. *BMJ*. 2010;340:c147.

9. Mease PJ, Gladman DD, Ritchlin CT, et al. Adalimumab for the treatment of patients with moderately to severely active psoriatic arthritis: results of a double-blind, randomized, placebo-controlled trial. *Arthritis Rheum*. 2005;52(10):3279-89.

10. Torii H, Nakagawa H, Japanese Infliximab Study i. Infliximab monotherapy in Japanese patients with moderate-to-severe plaque psoriasis and psoriatic arthritis. A randomized, double-blind, placebo-controlled multicenter trial. *J Dermatol Sci*. 2010;59(1):40-9.

11. Kavanaugh A, van der Heijde D, McInnes IB, et al. Golimumab in psoriatic arthritis: one-year clinical efficacy, radiographic, and safety results from a phase III, randomized, placebo-controlled trial. *Arthritis Rheum*. 2012;64(8):2504-17.

12. Ritchlin C, Rahman P, Kavanaugh A, et al. Efficacy and safety of the anti-IL-12/23 p40 monoclonal antibody, ustekinumab, in patients with active psoriatic arthritis despite conventional non-biological and biological anti-tumour necrosis factor therapy: 6-month and 1-year results of the phase 3, multicentre, double-blind, placebo-controlled, randomised PSUMMIT 2 trial. *Ann Rheum Dis*. 2014;73(6):990-9.

13. Kavanaugh A, Mease PJ, Gomez-Reino JJ, et al. Treatment of psoriatic arthritis in a phase 3 randomised, placebo-controlled trial with apremilast, an oral phosphodiesterase 4 inhibitor. *Ann Rheum Dis*. 2014;73(6):1020-6.

14. Kapiainen SV, Antti; Haula, Taru. Terveyden- ja sosiaalihuollon yksikkökustannukset Suomessa vuonna 2011. *Julkari*. 2014.

15. Rodgers M, Epstein D, Bojke L, et al. Etanercept, infliximab and adalimumab for the treatment of psoriatic arthritis: a systematic review and economic evaluation. *Health Technol Assess*. 2011;15(10):i-xxi, 1-329.

16. Torkki P, Leskela RL, Linna M, et al. Cancer costs and outcomes in the Finnish population 2004-2014. *Acta Oncol*. 2018;57(2):297-303.

17. Novartis. A phase III randomized, double-blind, placebo-controlled multicenter study of subcutaneous secukinumab in prefilled syringes to demonstrate the efficacy at 24 weeks and to assess the long term efficacy, safety and tolerability up to 5 years in patients wi. Protocol Number CAIN457F2312.

18. Burmester GR, Panaccione R, Gordon KB, et al. Adalimumab: long-term safety in 23 458 patients from global clinical trials in rheumatoid arthritis, juvenile idiopathic arthritis, ankylosing spondylitis, psoriatic arthritis, psoriasis and Crohn's disease. *Ann Rheum Dis*. 2013;72(4):517-24.

19. Conaghan PG, Alten R, Strand V, et al. The Relationship Between Physical Functioning and Work for People with Psoriatic Arthritis: Results from a Large Real-World Study in 16 Countries. *Arthritis Rheum*. 2016;68(Suppl 10).

20. Rodgers M, Epstein D, Bojke L, et al. Etanercept, infliximab and adalimumab for the treatment of psoriatic arthritis: a systematic review and economic evaluation. *Health Technol Assess*. 2011;15(10):i-xxi, 1-329.

21. Wong K, Gladman D, Husted J, et al. Mortality studies in psoriatic arthritis: results from a single outpatient clinic. I. Causes and risk of death. *Arthritis Rheum*. 1997;40(10):1868-72.

22. Abuabara K, Azfar RS, Shin DB, et al. Cause-specific mortality in patients with severe psoriasis: a population-based cohort study in the U.K. *Br J Dermatol*. 2010;163(3):586-92.

23. McInnes I, Nash P, Ritchlin C, et al. THU0437 Secukinumab for the treatment of psoriatic arthritis: comparative effectiveness results versus licensed biologics and apremilast from a network meta-analysis. *Annals of the Rheumatic Diseases*. 2016;75(2):348-9.
